# Effects of ω-3 Polyunsaturated Fatty Acids on Coronary Atherosclerosis and Inflammation: A Systematic Review and Meta-Analysis

**DOI:** 10.3389/fcvm.2022.904250

**Published:** 2022-06-20

**Authors:** Zheng Gao, Dewen Zhang, Xiaocan Yan, Hekai Shi, Xiaohui Xian

**Affiliations:** ^1^Department of Internal Medicine, The Second Affiliated Hospital of Hebei Medical University, Shijiazhuang, China; ^2^Department of Pathophysiology, College of Basic Medicine, Hebei Medical University, Shijiazhuang, China; ^3^Department of Pathophysiology, Hebei Key Laboratory of Critical Disease Mechanism and Intervention, Hebei Medical University, Shijiazhuang, China

**Keywords:** ω-3 polyunsaturated fatty acids, coronary atherosclerosis, endothelial inflammations, cardiovascular imaging, cardiovascular therapy

## Abstract

**Background and Purpose:**

Multiple guidelines suggest the ω-3 polyunsaturated fatty acids (ω-3 PUFAs) help to prevent major vascular events of coronary heart disease (CHD), but the data on large trials of ω-3 fatty acids are controversial. We reviewed the available evidence to determine the effect of ω-3 PUFAs on coronary atherosclerosis.

**Materials and Methods:**

Literature were from online databases. Randomized controlled trials (RCTs) or observational studies were acceptable. Quantitative data synthesis was conducted using R version 4.1.2. Each outcome was calculated using standardized mean difference (SMD) in a random-effect model. Sensitivity analysis was conducted for each outcome. A total of 21 RCTs and 1 observational study with 2,277 participants were included.

**Results:**

Meta-analysis indicated a benefit of ω-3 PUFAs on coronary atherosclerosis, namely, (1) ω-3 PUFAs can reduce the atherosclerotic plaque volume (SMD −0.18; 95% CI −0.31 to −0.05); (2) ω-3 PUFAs can help reduce the loss of the diameter of the narrowest segments of coronary arteries in patients with CHD (SMD 0.29; 95% CI, 0.05–0.53); (3) ω-3 PUFAs do not have significant effect on volume of lipid plaque in coronary arteries (SMD −1.18; 95% CI −2.95 to 0.58), volume of fiber plaque (SMD 0.26; 95% CI −0.81 to 1.33), and calcified plaque (SMD 0.17; 95% CI −0.55 to 0.89); and (4) ω-3 PUFAs had no significant effect on endothelial inflammatory factors in peripheral blood.

**Conclusions:**

We confirmed that ω-3 PUFAs benefit patients with CHD by reducing the progression of coronary atherosclerosis. We indicated that the benefits were not caused by reducing endothelial inflammations of coronary arteries.

**Systematic Review Registration:**

https://www.crd.york.ac.uk/prospero/display_record.php?ID=CRD42021285139, identifier: CRD42021285139.

## Graphical Abstract

In order to increase the exposure of our work, we made a graphical abstract: ω-3 PUFAs (ω-3 polyunsaturated fatty acid) reduce coronary atherosclerotic plaques growth and relieve the lesions process of the most stenotic segment of the coronary artery. However, the effects on all main compositions of atherosclerotic plaques (fiberboard, calcified plaque, and lipid plaque) are not statistically significant. Serum soluble vascular cell adhesion molecule-1 (sVCAM-1) level and activation percentage of von Willebrand factor (VWF%) are not significantly affected. The mechanism of ω-3 PUFAs on coronary atherosclerosis may need further studies. 
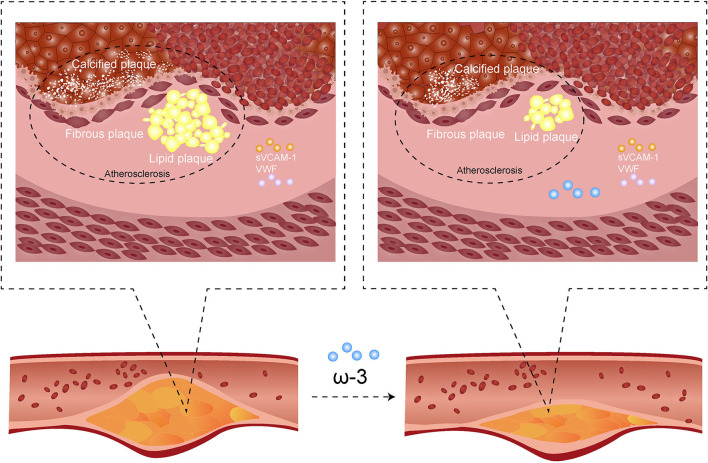

## Introduction

Omega-3 polyunsaturated fatty acids (ω-3 PUFAs) are a class of PUFAs with the first unsaturated double bond between the third and fourth carbon atoms from the methyl end. ω-3 PUFAs have various effects on the cardiovascular system and can provide multiple health benefits for the cardiovascular system (1, 2) *via* various mechanisms, such as reduction in blood lipids and inflammatory cell aggregation (3–10), and the reduction of triglycerides in the blood is thought to be the main benefit (11). Multiple studies have investigated the association between ω-3 PUFAs supplementation and less coronary atherosclerotic “high-risk” plaque. In fact, atherosclerotic plaques can be mainly classified into four different types, namely, calcified tissue, necrotic or soft tissue, fibrous tissue, and mixed tissue (12, 13). In recent years, many studies tried to determine whether ω-3 PUFAs have a protective effect on coronary atherosclerosis in patients with coronary heart disease (CHD). However, the results among different studies were controversial. For example, Balk et al. (14) reported that ω-3 PUFAs showed prevented effect on restenosis after percutaneous transluminal coronary intervention (PCI). In contrast, Laake et al. (15) suggested that ω-3 PUFAs had no significant clinical effect on CHD. Previous meta-analyses only investigated the relation between ω-3 PUFAs supplementation and major vascular events of CHD. However, there is a lack of information focusing on the effect of ω-3 PUFAs on the pathology of plaques and inflammation level. Therefore, this meta-analysis systematically summarized and analyzed the effects of ω-3 PUFAs on coronary atherosclerosis and inflammation level of vascular endothelial.

## Methods

This meta-analysis was performed following the Preferred Reporting Items for Systematic Reviews and Meta-analyses statement (16) and Cochrane's guidelines (17). It has been registered in the international Prospective Register Of Systematic Reviews (CRD42021285139). The review protocol can be accessed at https://www.crd.york.ac.uk/PROSPERO/. We strictly followed the protocol while conducting this review. All the included studies were confirmed to be free of ethical and moral concerns. Data for the review were analyzed anonymously. So, the need for consent was waived by the ethics committee.

### Search Strategy and Selection Criteria

Three reviewers searched articles from Embase database, Web of Science, PubMed, Cochrane Library, Clinical Trial, and China National Knowledge Infrastructure (CNKI) (published from February 1979 to September 2021) for published randomized clinical trials (RCTs) and controlled clinical trials, aiming to assess the effect of ω-3 PUFAs on coronary atherosclerosis in patients with CHD. We used atherosclerotic plaques, ω-3 polyunsaturated fatty acids, vessels, blood, fibroatheroma, and coronary heart diseases as the keywords for the literature search.

**Figure 1:**
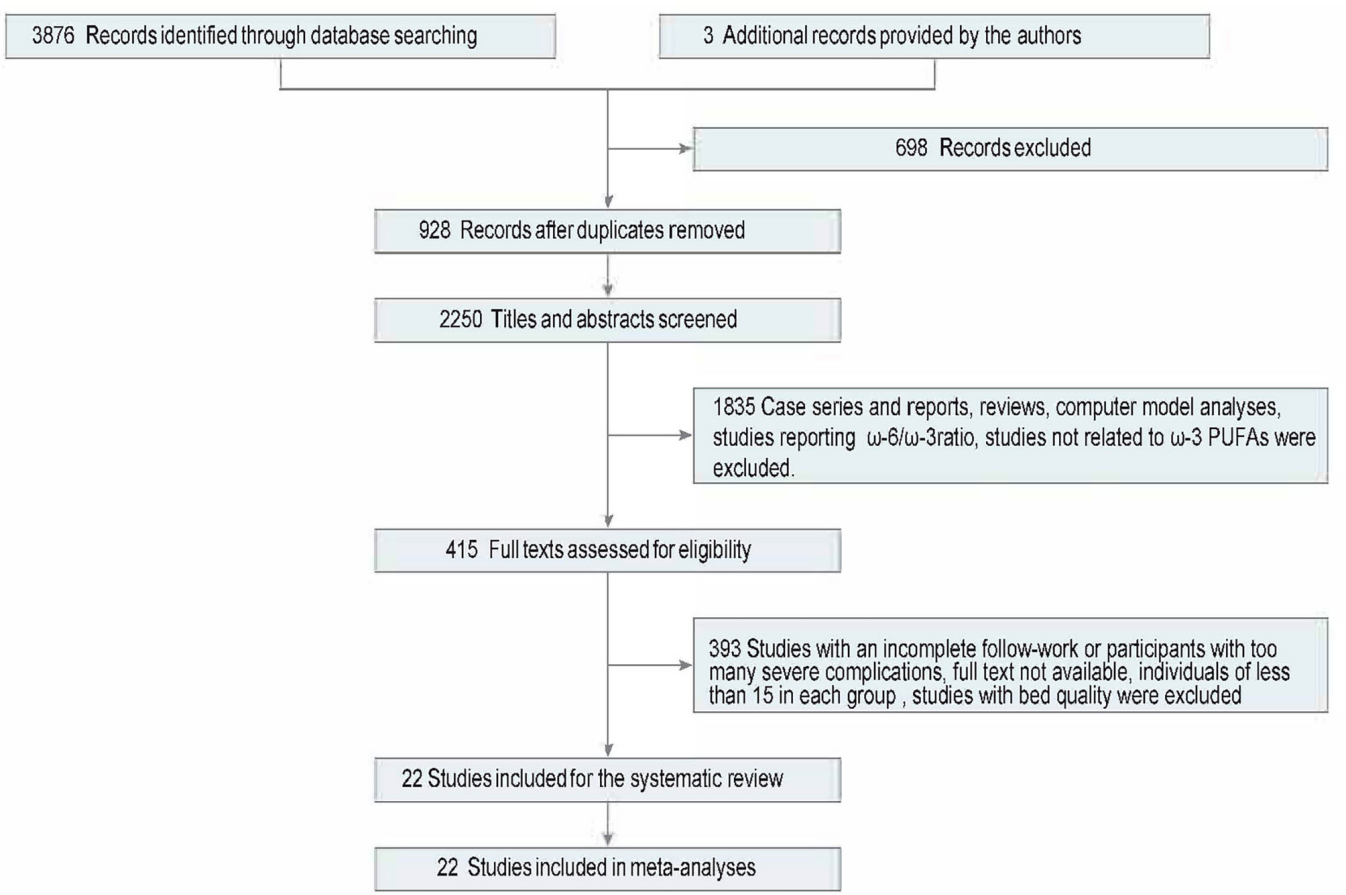
Flowchart of literature identifying.

The eligibility criteria were (1) interventions for RCTs should be eicosapentaenoic acid (EPA) + docosahexaenoic acid (DHA) at a total dose of no <1 g/day, as recommended to patients with CHD (18) since ω-3 PUFAs are mainly represented by EPA and DHA (19–21). (2) The subjects for coronary imaging should be patients diagnosed with CHD or those with a high risk of CHD determined by the original clinical researchers. The subjects should not include patients with multiple complications; for example, severe diabetes, heart failure, arrhythmia, hypertrophic cardiomyopathy, and cardiac syndrome X and kidney disease would be excluded. (3) All the data needed can be extracted from the research article. (4) Death cases were excluded from endpoint assessment. (5) We included only studies that all the follow-up work completed to ensure the whole data was available. (6) The number of follow-up patients should not be <15, which means the data could be calculated by Cohen's *d* effect size. (7) Studies on endothelial cell markers could include healthy individuals but with no platelet dysfunction. Studies of bed quality, those with inadequate sample size, incomplete follow-up works, or studies that did not meet the criteria were excluded. Disagreement in literature identifications was reported to another reviewer. The search strategy is provided in Figure 1; Supplementary Appendix 1.

The primary outcomes were (1) the volume increases in coronary atherosclerotic plaques and (2) reduction in the diameter of the narrowest segments of the coronary arteries. The secondary outcomes consisted of endothelial inflammatory factor levels in the peripheral blood [including activation of von Willebrand factor (VWF%) and content of soluble vascular cell adhesion molecule 1 (sVCAM-1)] and compositions of plaques (fiber plaque, lipid plaque, and calcified plaque).

#### Selection of Studies

Following the inclusion and exclusion criteria, three researchers (ZG, XY, DZ) screened, selected, and extracted data from studies independently. All the data should be extracted directly from the articles, in the tables or text. Data from images were not contained in this review. Any disagreement was reported to an experienced doctor (HS) to decide whether to include the literature. One reviewer (ZG) conducted data synthesis for all trials. Screening and selection of included trials are shown in a flow diagram (Figure 1).

#### Data Extraction and Management

Three authors (GZ, YXC, and ZDW) independently extracted clinical variables and outcome data using the retrieval format of population, intervention, control, and outcomes (PICO), i.e., (1) basic information, including publishing year, authors, country, and journal; (2) population: age, gender, complications, country, body mass, history of heart surgery, history of other medications use like stains, and blood lipid levels. We excluded studies with (3) interventions: placebos, type of ω-3 PUFAs, treatment duration of ω-3 PUFAs. (4) Outcome: volume of coronary atherosclerotic plaques and their compositions, loss of diameter of the narrowest segments of the coronary arteries, the content of sVCAM-1, and VWF%. (5) Designs: intergroup or intragroup RCT.

#### Assessment of Risk of Bias

All included trials were assessed for risk of bias using the following quality scales: (1) bias arising from the randomization process; (2) bias due to deviations from intended interventions; (3) bias due to missing outcome data; (4) bias in measurement of the outcome; and (5) bias in selection of the reported result. Each potential source of bias was graded into three levels, namely, “low,” “some concerns,” and “high.” The quality assessments were performed using R software (version 4.1.1) under the guidance of the Cochrane Handbook (17) and RoB2 (22), the revised Cochrane risk-of-bias tool for randomized trials.

#### Data Analysis

Heterogeneity among studies was estimated using Cochran's *Q* test and quantified by the *I*^2^ statistic. Each outcome was calculated using a standardized mean difference (SMD) in a random effect model. *I*^2^ values of 25, 50, and 75% were thought to indicate a low, moderate, or high heterogeneity (23). We used Cohen's *d* as the effect for *p-*values and 95% confidence intervals. Statistical significance was determined using a two-sided α < 0.05. Funnel plots and peter's tests were employed to estimate publication bias. Meta-regression analyses were used to detect possible sources of heterogeneity. Potential publication biases were further determined by the function “trim and fill.” Publication bias was considered significant when the trimmed result led to an inconsistent conclusion. Sensitivity analysis was performed to determine heterogeneity and individual studies' influence on overall estimates. Studies were serially excluded using the function “metainf” for the sensitivity analyses. The included studies in each group were excluded one by one in the method. All data analyses were conducted using R version 4.1.2, using the package “meta” (R Project for Statistical Computing) (R Core Team. R: a language and environment for statistical computing. Vienna R Foundation for Statistical Computing; 2019. https://www.R-project.org).

The results are described based on “Per-Protocol analyses” (24). For the studies available, we adjusted for factors, such as age and body mass index. For outcomes with significant heterogeneity, meta-regression analyses were used to detect possible sources of heterogeneity. We mainly focused on the average age of the population and the duration of follow-up, which were believed to be major factors affecting the effects of medicines.

## Results

### Description of Studies

A flow diagram of this systematic review is shown in Figure 1. Studies that had not met the inclusion criteria mentioned above were excluded. The literature search yielded 3,879 studies, of which 22 studies (24–45) with 2,277 participants were included in this systematic review and meta-analysis (Table 1). Of the 22 included studies, 21 (24–31, 33–45) were RCTs and 1 was an observational study (32) (Table 1). All follow-up work was completed when studies were identified.

**Table 1:** Characteristics of studies.

**References**	**Year**	**Characteristic in baseline**						**Intervention**		**Outcome measures**	**Follow-up time**
		**Omega-3**			**Control**					
		**Age**	**Sex, Male *n* [%]**	***N***	**Age**	**Sex, Male *n* [%]**	***N***	**Omega-3**	**Control**		
Abdulhamied Alfaddagh (24)	2017	62.6 ± 7.5	84 [86.6%]	122	62.4 ± 7.8	104 [85.2%]	97	DHA	Placebo	c.d.e.f.	30-Month
Abdulhamied Alfaddagh (35)	2019	63.5 ± 7.8	110 [82.7%]	133	62.1 ± 7.7	72 [84.7%]	85	DHA	Placebo	d.e.f.	30-Month
Arja T. Erkkilä (45)	2006	*N* ^a^	*N*	114	*N*	*N*	114	EPA and DHA	Placebo	i.	3.2 ± 0.6 Years
Clemens von Schacky (42)	1999	57.8 ± 9.7	91 [82.0%]	29	58.9 ± 8.1	88 [78.6%]	29	DHA	Placebo	i.	2 Years
Elsa M Hjerkinn (36)	2005	70 [64-76]	*N*	124	70 [64-76]	*N*	119	EPA and DHA	Placebo	g.i.	3 Years
F. M. Sacks (37)	1995	62 ± 7	29 [93.5%]	179/31^b^	62 ± 7	26 [92.9%]	126/28^c^	DHA	Placebo	i.	2.4 Years
Matthew J. Budoff (38)	2020	56.5 ± 8.9	17 [54.8%]	31	58.3 ± 8.6	20 [54.1%]	37	IPE	Placebo	c.d.e.f.	18–24 Months
O. Johansen (39)	1999	57.3 [43-74]	18 [78.3%]	23	57.7 [40-73]	21 [67.7%]	31	DHA	Placebo	g.i.	6 Months
S. L. Seierstad (40)	2005	58 [46-74]	17 [85%]	20	63 [52-75]	18 [94.7%]	20	DHA	Placebo	g.	NA
Tetsu Watanabe (41)	2017	67 ± 10	78 [80%]	97	68 ± 10	81 [84%]	96	EPA	Placebo	c.d.e.f.	6–8 months
Toshiyuki Niki (44)	2016	68.1 ± 10.1	21 [72%]	29	69.4 ± 10.7	19 [63%]	30	EPA	Placebo	c.d.e.f.	6 months
I. Seljeflot (43)	1998	49.5 [41-57]	*N*	22	48 [70-112]	*N*	19	DHA	Placebo	g.i.	6 weeks
Jinhee Ahn (25)	2016	59.6 ± 9.1	24 [63.2]	38	60.7 ± 0.8	26 [72.2]	36	EPA and DHA	Placebo	d,i.	12 Months
Yoichiro Sugizaki (34)	2020	70.8 ± 7.7	17 [81.0]	21	75.3 ± 8.8	16 [76.2]	21	EPA+Rosuvastatin	Placebo	i.	12Months
Yoko Kita (33)	2020	63 [55-73]	27 [87]	31	63 [55-73]	24 [77]	31	EPA	Placebo	d.	8 Months
Tetsuya Amano (32)	2011	70 ± 10	82 [43]	58	70 ± 10	82 [43]	58	EPA <1.9	EPA≥1.9	d,e,g,i	NA
Leah E. Gillingham (27)	2011	*N*	*N*	36	*N*	*N*	36	Flaxseed	High-oleic rapeseed oil diet	g.	2 Years
M. Mirfatahi (29)	2016	68.0 ± 3.0	12.0 [71.0%]	17	59.0 ± 4.0	10.0 [59.0%]	17	Flaxseed oil	Placebo	g.	8 Weeks
Paula Berstad (30)	2003	70 ± 3	*N*	47	70 ± 3	*N*	42	Dietary + VLC n-3	Dietary intervention	g.	3 Years
Kaeng W. Lee (26)	2005	59 ± 10	35 [95]	37	55 ± 10	36 [96]	40	Omacor	Usual care	i.	3 Month
Melinda Phang (28)	2014	*N*	*N*	31	*N*	*N*	32	EPA and DHA	Placebo	i.	4 Weeks
Szu-Yun Wu (31)	2014	*N*	*N*	84	*N*	*N*	84	DHA	Placebo	g	8 Weeks

*Data are expressed in mean ± SD or interquartile range.

*^a^N means none*.

*^b^Number of samples of lesions/population size*.

*^c^Associations of ω-3 PUFAs supplementation and coronary atherosclerotic plaques*.

*^d^Change in volume of the lipid plaques*.

*^e^Change in volume of the fiber plaques*.

*^f^Change in volume of the calcified plaques*.

*^g^Content of sVCAM-1 in peripheral blood*.

*^h^Content of von Willebrand factor in peripheral blood*.

*^i^The diameter of the narrowest segment of Cas*.

**Figure 2:**
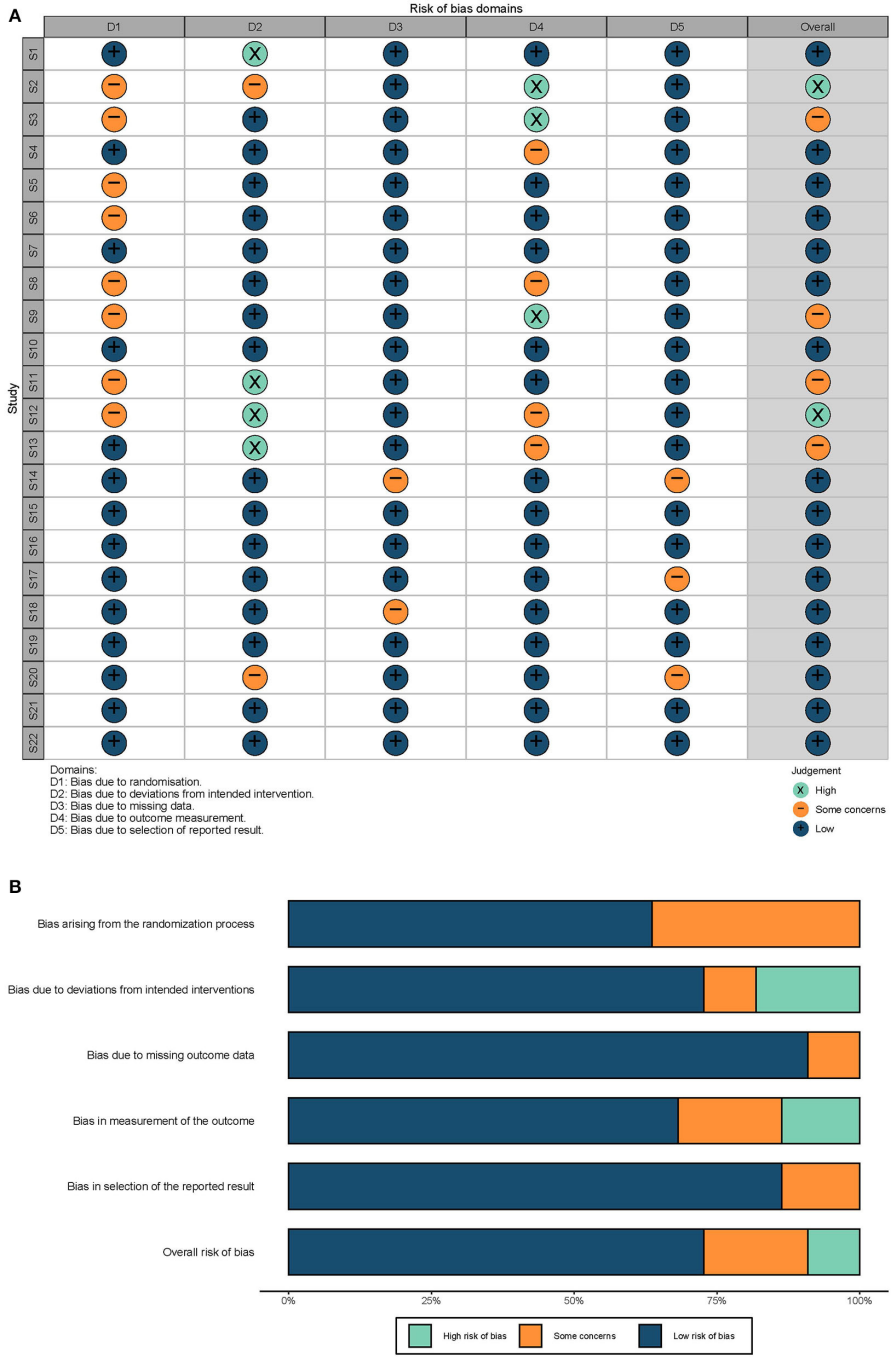
Quality evaluations. **(A)** Risk biases of each study. S1–S22: 22 studies included in this review, serially. The overall risk was evaluated based on the criteria described previously. **(B)** Summary of risk biases. The length of each color represents the proportion of the risk.

Of the 22 studies, 2 (9.09%) were at a high risk of bias, 4 (18.18%) were at “some concerns,” and 16 (72.73%) were at low risk of bias (Figure 2). Studies with one domain of high risk and only one domain of “some concerns” were thought to be at “some concerns.” Studies with one “high-risk” domain and two or more domains of “some concerns” were thought to be at “high risk.” Studies with two or more domains of “high risk” were excluded. A comprehensive assessment of the included studies suggests the present evidence of well credibility.

The mean age of individuals included in the studies was over 50 years old except for one (40), if characteristics were available. All available studies enrolled mainly male participants. Placebos was used in 19 studies (24, 25, 27, 29, 31–45) as the controls, and dietary interventions were used in 3 (26, 28, 30) studies. One observational study (32) used EPA index of 1.9 as the basis for dividing observation and control group. Plaque volume in related studies was measured using imaging techniques, including coronary computed tomographic angiography (24, 35, 38), optical coherence tomography (33, 34), and intravascular ultrasound (25, 32, 38, 41, 44). Characteristics of the included studies were extracted by one of us (GZ), which are described in a table (studies are shown in Table 1). As shown in each forest plots, three studies were conducted in England (26, 28, 31), one in Korea (25), one in Canada (27), five in Japan (32–34, 41, 44), five in Norway (30, 36, 39, 40, 43), one in Iran (29), one in Germany (42), one in Finland (38), three in Israel (24, 35, 37) and one conducted in Finland and the United States (45).

### Data Synthesis

#### Primary Outcomes

##### Associations of ω-3 PUFAs and Coronary Atherosclerotic Plaques

**Figure 3:**
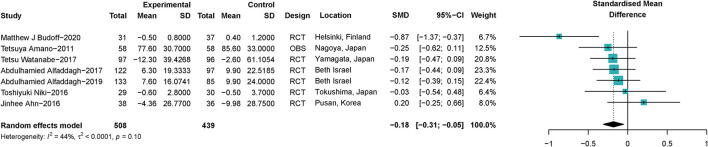
Forest plots of associations of ω-3 PUFAs supplementation and coronary atherosclerotic plaques. Point sizes are an inverse function of the precision of the estimates, and bars correspond to 95% CIs. Data are calculated using a random-effect model. RCT, randomized controlled trial; OBS, observational study.

A total of seven trials (24, 25, 32, 35, 38, 41, 44) with 947 participants investigated the correlation between ω-3 PUFAs and sizes of coronary atherosclerotic plaques. It was shown that ω-3 PUFAs could reduce the atherosclerotic plaque volume (SMD −0.18; 95% CI −0.31 to −0.05), with a low heterogeneity (*I*^2^ = 44%) (Figure 3). Omega-3 PUFAs' effect was significantly protective on atherosclerotic plaques when Niki's et al. (44) study or Ahn's et al. (25) study was excluded, same as shown in the sensitivity analysis (Supplementary Figure S1.1). Heterogeneity did not reduce significantly when studies were excluded serially (Supplementary Figure S1.2). We further explored whether the effect of ω-3 PUFAs supplementation on plaque volume was dose-related. The linear regression showed that there was no significant correlation between the dose of ω-3 PUFAs supplementation and plaque volume change (*R*^2^ = 0.09, *p* = 0.29), same as shown in Supplementary Figures S1.3, S1.4.

##### Associations of ω-3 PUFAs Supplementation and Most Stenotic Segment of the Coronary Artery

**Figure 4:**
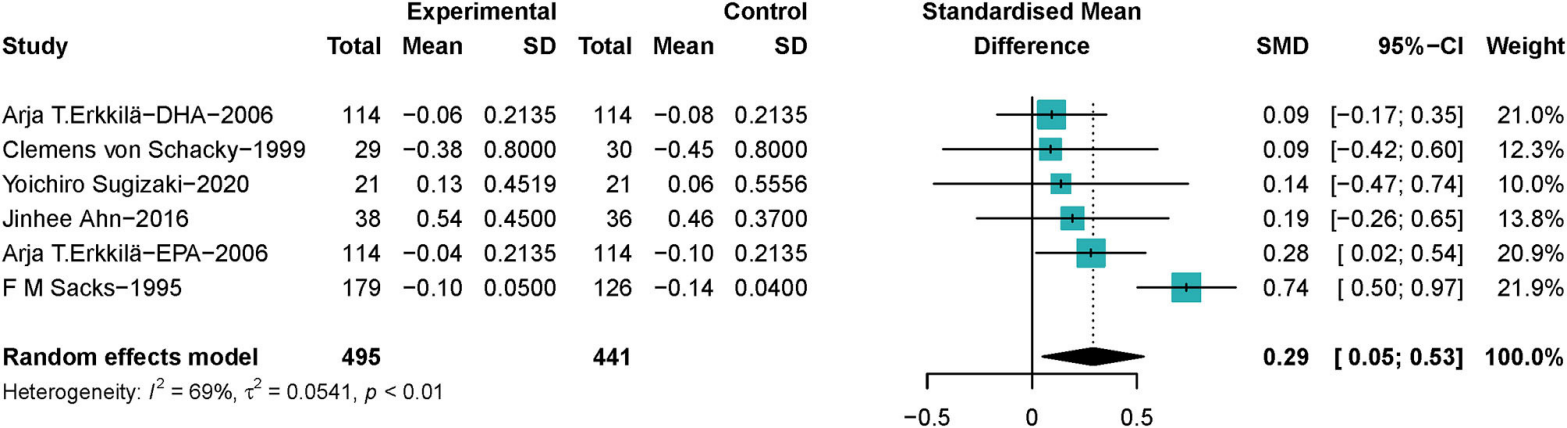
Forest plots of associations of ω-3 PUFAs and most stenotic segment of the coronary artery. Point sizes are an inverse function of the precision of the estimates, and bars correspond to 95% CIs. Data are calculated using a random-effect model.

A total of 936 lesions samples from 462 patients with CHD were included to investigate the association between ω-3 PUFAs and stenosis of the coronary artery, in which 233 patients with CHD received ω-3 PUFAs supplements, and 229 patients received placebos. One study (45) provided data on both EPA and DHA, which we took into account. The pooled data showed that ω-3 PUFAs could help reduce the loss of the diameter of the narrowest segments of CA in patients with CHD (SMD 0.29; 95% Cl 0.05–0.53), with a moderate heterogeneity (*I*^2^ = 69%) (Figure 4). ω-3 PUFAs' effect became not significant on the most stenotic segment of the coronary artery when the study Erkkilä et al. (45) was excluded, as shown in Supplementary Figure S2.1. Heterogeneity became 0% when Sacks' et al. (37) study was excluded (Supplementary Figure S2.2).

#### Secondary Outcomes

##### Plaque Compositions

**Figure 5:**
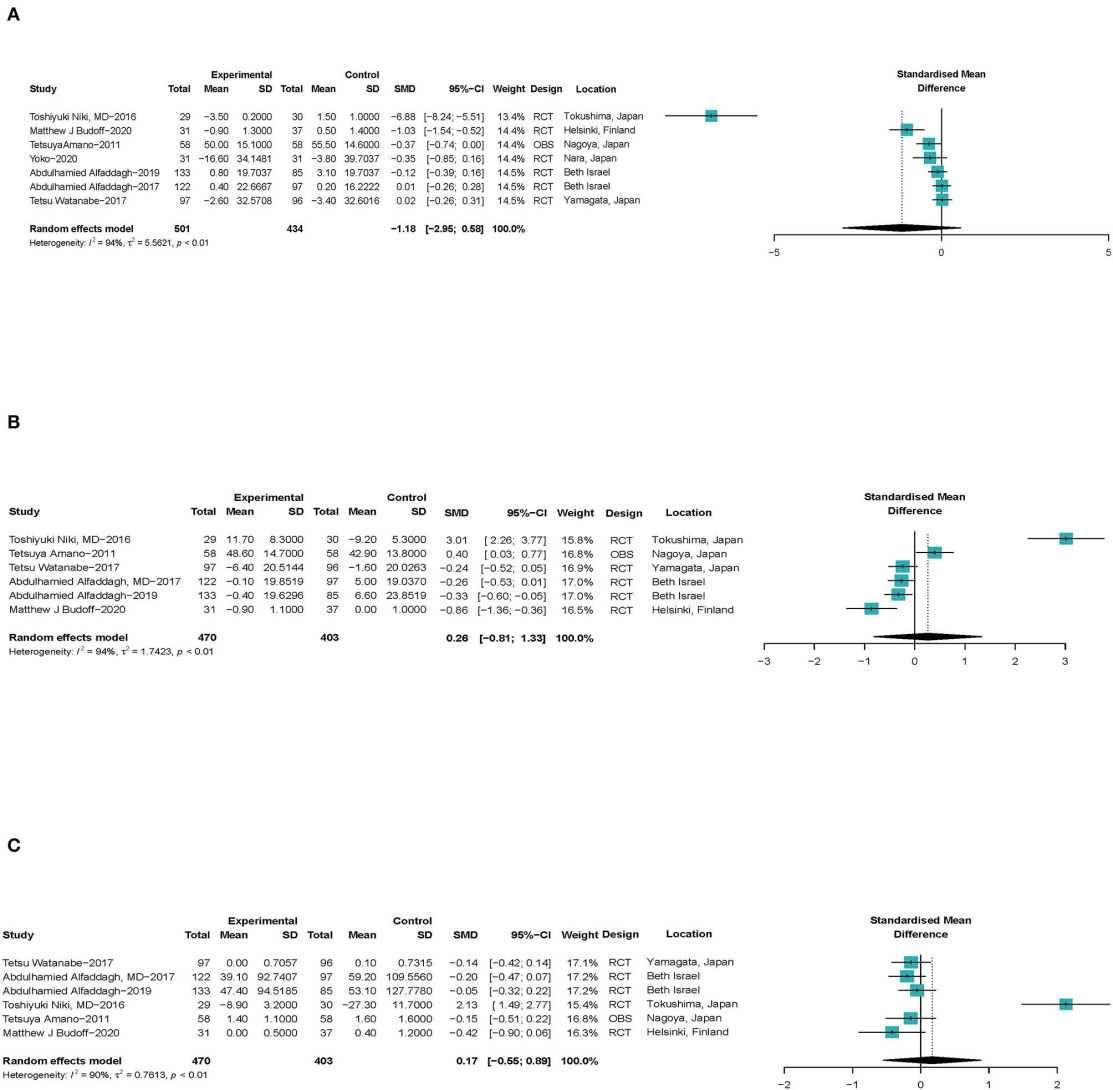
Forest plots of associations of ω-3 PUFAs and coronary atherosclerotic plaque compositions. **(A)** Lipid plaque volume, **(B)** fiber plaque volume, and **(C)** calcified plaque. Point sizes are an inverse function of the precision of the estimates, and bars correspond to 95% CIs. Data are calculated using a random-effect model. RCT, randomized controlled trial; OBS, observational study.

As shown in Figure 5, seven studies (24, 32, 33, 35, 38, 41, 44) with a total of 935 patients with CHD reported the effect of ω-3 PUFAs on the volume change of lipid plaque in coronary arteries (SMD −1.18; 95% CI −2.95 to 0.58) with a significant heterogeneity (*I*^2^ = 94%). The outcome was stable when studies were excluded serially (Supplementary Figure S3.1). Heterogeneity reduced significantly when Niki's (44) study was excluded (Supplementary Figure S3.2). For Amano's study, we only chose EPA to represent ω-3 PUFAs, which was observed to be significantly different between the acute coronary syndrome (ACS) and non-ACS groups in Amano's participants. It was believed that the conclusions drawn in this way may be more clinically meaningful. Associations between ω-3 PUFAs and volume of fiber plaque were reported by six studies (24, 32, 35, 38, 41, 44) (SMD 0.26; 95% CI −0.81 to 1.33), with a significant heterogeneity (*I*^2^ = 94%), same as shown in Figure 5. Both the estimates and heterogeneities were stable when studies were excluded serially (Supplementary Figures S4.1, S4.2). As shown in Figure 5, six studies (24, 32, 35, 38, 41, 44) with a total of 873 patients with CHD reported the effect of ω-3 PUFAs on the volume change of calcified plaque in coronary arteries (SMD 0.17; 95% CI −0.55 to 0.89) with a significant heterogeneity (*I*^2^ = 90%). The exclusion of Niki's et al. (44) study led to both a significant prospective effect of ω-3 PUFAs on calcified plaque volume and a very low heterogeneity (Supplementary Figures S5.1, S5.2).

Outcomes suggest that ω-3 PUFAs have no significant effect on the volume of atherosclerotic plaque compositions.

##### sVCAM-1 and VWF%

**Figure 6:**
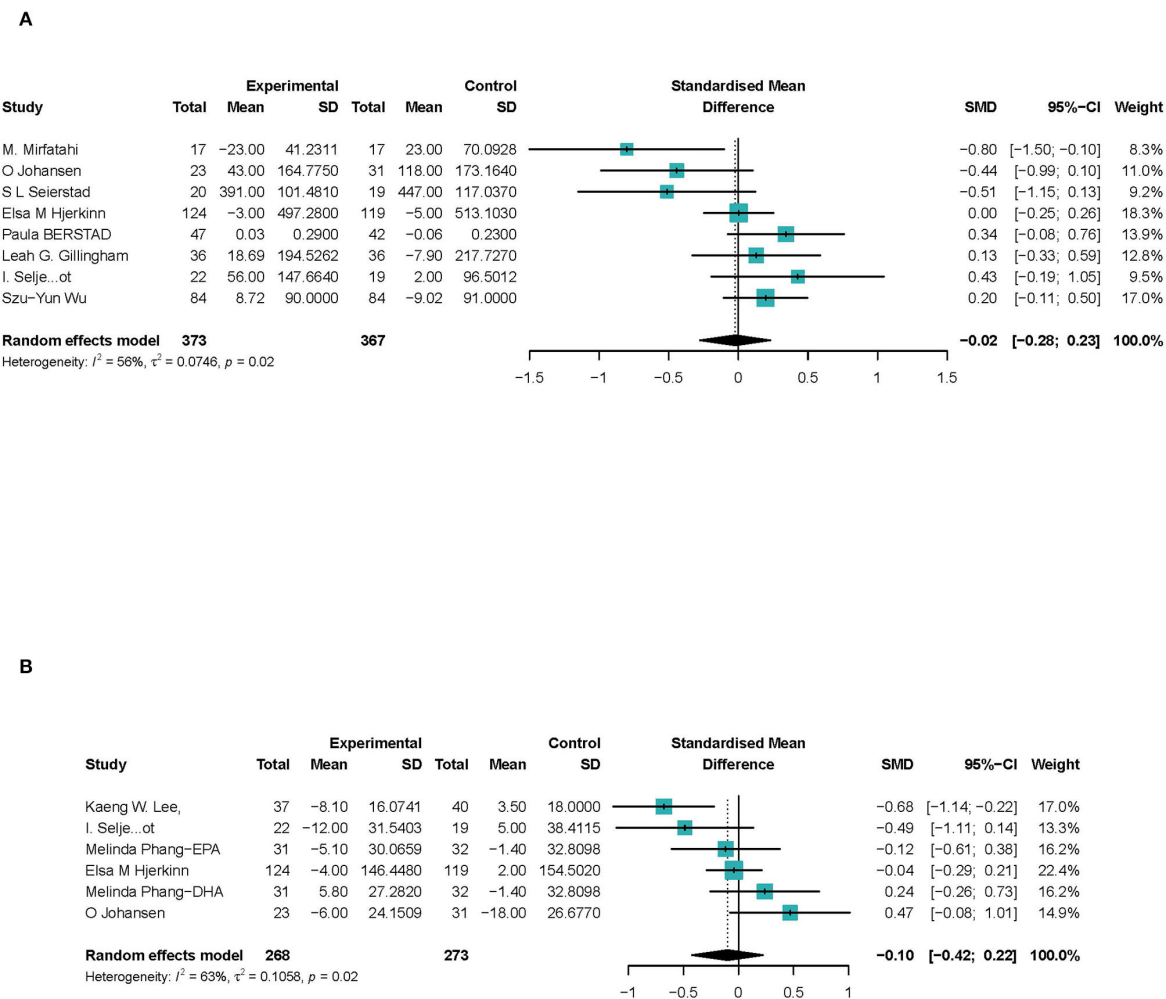
Forest plots of associations of ω-3 PUFAs and endothelial cell markers. **(A)** sVCAM-1; **(B)** VWF%. Point sizes are an inverse function of the precision of the estimates, and bars correspond to 95% CIs. Data are calculated using a random-effect model.

In eight studies (27, 29–31, 36, 39, 40, 43), a total of 740 participants were included to investigate the association between ω-3 PUFAs and sVCAM-1 level in peripheral blood. The results showed that ω-3 PUFAs had no significant effect on sVCAM-1 in peripheral blood (SMD −0.02; 95% Cl −0.28 to 0.23), with a moderate heterogeneity (*I*^2^ = 54%) (Figure 6). Outcomes were stable when studies were excluded serially, heterogeneity significantly reduced when Mirfatahi's study (29) was excluded (Supplementart Figures S6.1, S6.2).

Six studies (26, 28, 36, 39, 43, 45) focus on the activation of VWF (VWF%). One study (28) reported both EPA and DHA's effect on activation of VWF, which we discussed, respectively. A total of 478 CHD participants were included, and 541 were calculated to investigate the association between ω-3 PUFAs and VWF activation in peripheral blood. The results showed that ω-3 PUFAs had no significant effect on VWF% (SMD −0.10; 95% Cl −0.42 to 0.22), with a moderate heterogeneity (*I*^2^ = 63%) (Figure 6). Estimates were stable when studies were excluded, and heterogeneity reduced significantly when Lee's et al. (26) study was excluded (Supplementary Figures S7.1, S7.2).

In summary, we suggested that ω-3 PUFAs have no significant effect on endothelial inflammatory factors in peripheral blood.

### Publication Bias

Publication bias was detected in the outcome on lipid plaque volume. The trimmed result led to a consistent conclusion, which suggested a negative publication bias, as shown in Supplementary Figures S8.1–S8.3. Publication bias was not found in other outcomes, as shown in funnel plots and Peter's tests (Supplementary Figures S9.1–S9.6, S10.1–S10.6). Pooled results of publication bias tests, sensitivity analyses, and quality assessments suggest conclusions of the present meta-analysis of high quality. The standardized mean difference, sensitivity analysis, heterogeneity and publication bias (Peter's test) for each outcome in the present review were summarized in Table 2.

**Table 2:** Summary of findings.

**Outcomes**	**SMD**	**(95%CI)**		***I*^2^ (%)**	**Peter's test**		**Sensitivity interval**	
					***T*-value**	***P*-value**		
Most stenotic segment of the coronary arteries	0.29	0.05	0.53	69%	1.74	0.1576	0.1742	0.3415
VWF%	−0.1	−0.42	0.22	63%	−0.16	0.8797	−0.191	0.0122
sVCAM-1	−0.02	−0.28	0.23	54%	−1.18	0.2828	−0.1564	0.2814
Effect on calcified plaques volume	0.17	−0.55	0.89	90%	1.69	0.1663	−0.1564	0.2814
Effect on volume of coronary atherosclerosis plaques	−0.18	−0.31	−0.05	44%	−0.4	0.7036	−0.2127	−0.1314
Effect on fiber plaques volume	0.26	−0.81	1.33	94%	−1.02	0.3645	−0.2389	0.4765
Effect on lipid plaques volume	−1.18	−2.95	0.58	94%	−5.58	0.0025	−0.2553	−1.3775

*SMD, standardized mean difference; CI, confidence interval; sensitivity interval: the range of SMD variation in the sensitivity analysis; T, effect size of Peter's test. Peter's test is used for the evaluation of publication bias*.

The results of meta-regression failed to confirm the influence of the average age of the population or the length of follow-up on the results. The quantitative results of meta-regression are described in Supplementary Figures S11.1–S11.12. The meta-regression curves are described in Supplementary Figures S12.1–S12.12.

## Discussion

This study is based on 22 studies, which included 2,277 participants. The large evidence base, obtained through multiple databases, provided us with more information on the effect of ω-3 PUFAs in CHD pathology. Our data showed that ω-3 PUFAs significantly slowed the atheromatous plaque volume increases of coronary arteries.

To the best of our knowledge, this meta-analysis was the first study that summarized the effect of ω-3 PUFAs on coronary atherosclerosis and analyzed potential mechanisms. We first explored the potential effect of ω-3 PUFAs on lipid plaque, fibrous plaque, calcified plaque, and two important endothelial inflammatory factors (sVCAM-1 and VWF). Our data showed that ω-3 PUFAs had an effect on the plaque growth in coronary arteries of patients with CHD, which was consistent with other studies. Previously, many studies have proved the efficiency of ω-3 PUFAs on the prevention of major vascular events, with an unclear mechanism (46–50). We suggested that ω-3 PUFAs may slow the growth of plaque in coronary arteries and then delay the progression of atherosclerosis. The effect of ω-3 PUFAs supplementation on plaque volume was not dose-related. We indicated that the chief mechanisms may not contain ω-3 PUFAs' effect on lipid, fibrous, or calcified compositions or endothelial inflammatory factors. Moreover, previous reports had suggested that the outcomes of ω-3 PUFAs supplementation may vary by patients' prior medications, such as stains (51). However, this meta-analysis demonstrated no heterogeneity in the effects of ω-3 PUFAs on plaque size of patients with CHD with/without the use of stains. Besides, we investigated the effect of ω-3 PUFAs on the lumen of coronary arteries. The data showed that ω-3 PUFAs contribute to the changes of the lumen of coronary arteries. The full-text review of the literature showed that in a considerable number of patients, even if ω-3 PUFAs reduced the progression of some lesions, the overall lesions were still deteriorating. We suggested that atherosclerosis is an inevitable disease of aging, contributed by various pathological changes, and the effect of ω-3 PUFAs might be limited.

Several studies have reported that ω-3 PUFAs can cause changes in arterial intimamedia thickness (52, 53), which may also be associated with the changes in the lumen of coronary arteries.

In patients with CHD, sVCAM-1 is closely related to atherosclerosis lesions in the early stage (54–56), in stable and unstable angina and acute myocardial infarction, by accelerating mononucleosis accumulates into endothelial cells (57). Von Willebrand factor is another important marker for the inflammatory activity of the vascular endothelium (58–60), especially activation rate (VWF%). Receptors and cytokines that participated in the process may include G protein-coupled receptors 120, cyclooxygenase-2, induced nitric oxide synthase 2 (61), interleukin 1 beta, and interleukin 6 (62). Outcomes on VWF% and content of sVCAM-1 both suggested that ω-3 PUFAs did not reduce the inflammatory responses of peripheral vessels.

Fiber plaque is also one of the major compositions of coronary atherosclerotic plaque that consists of a large number of collagen fibers, a few elastics and proteoglycans, foam cells, extracellular lipids, and inflammatory cells (12). Lipid pool and granulation tissue reaction could only be seen in the late stage of the lesion (63). This suggests that collagen fibers are the main component of fiber plaques. The volume of collagen fibers is mainly affected by inflammatory factors and inflammatory cells (64). As mentioned earlier, inflammatory activity was not significantly affected by ω-3 PUFAs. This explains why the volume of fibrous plaques is not affected by ω-3 PUFAs. Therefore, we confirmed that the change of fiber plaque volume is not one of the main causes for the reduction of growth of atherosclerotic plaques. The formation of calcified plaques is considered to be due to the accumulation of crystalline calcium in the lipid bodies of the nucleus. Some scholars reported that sheet calcification is highly prevalent in stable plaques, while microcalcifications, punctate, and fragmented calcifications are more frequent in unstable lesions, which means calcification of different degrees may play a dual role in the stability of coronary atherosclerotic plaques (65). This suggests that the result of the current meta-analysis on calcification plaques may be due to differences in calcification in different populations.

Previous studies have demonstrated that the formation of plaques plays a key role in the pathology of CHD (66). Thrombotic lesions are composed of a fibromuscular cap overlying a large necrotic tissue core containing macrophages and lipid core. However, there exist other plaques that contain more fibrotic tissues, and these types of plaques are likely to rupture and generate thrombosis. Therefore, depending on plaque composition, coronary artery disease may predispose to stable angina or unstable angina (67). We fail to suggest whether ω-3 PUFAs benefit more to patients with stable or unstable angina.

A previous study reported the controversial role of ω-3 PUFAs on cardiovascular events in severe and lethal cardiovascular events—some reported protective effects (46–50)—while others reported no significant effects (68, 69). Our review explained the effect of ω-3 on segments of severe lesions at a more microscopic level. By summarizing the data, we found that ω-3 PUFAs reduced the lesion of the narrowest segments, which supported the former conclusion mentioned above. Studies have shown that regular intake of both EPA + DHA as supplements may bring benefits for patients with CHD through reduction of arrhythmias, endothelial dysfunction, and inflammation (70, 71). This review actually challenges the inflammatory mechanism. Safi's et al. (46) review also gave an interpretation that EPA monotherapy brings more profiles for patients with CHD than treatment with EPA + DHA, and the physiological mechanism cannot be reflected in this review.

Heterogeneity was generally moderate among the measures in this review. We analyzed the heterogeneity through sensitivity analyses and detected several potential sources. We further full-text reviewed the included studies. The possible sources of heterogeneity are summarized as follows. (1) Statistical heterogeneity: some studies use a quartile spacing table, while others use mean and standard deviation to represent the outcomes, which may generate statistical heterogeneity in the process of merger and transformation. (2) Standardized patients are rarely used in the existing interested studies, and different ethnic populations and those with comorbidities may produce heterogeneity. We conducted a sensitivity analysis for impact factors of each outcome and provided the results in Supplementary Figures S5.1–S5.10. (3) Heterogeneity may result from different designs of the studies, including the length of follow-up and the criteria of the population included in the follow-up measurement, for the reason that the progression of the lesions may vary in different populations with different duration. However, the results of meta-regression failed to confirm the influence of the average age of the population or the length of follow-up on the results. (4) The imaging methods and kits used in the evaluation of coronary atherosclerosis and the measurement of endothelial factors vary greatly.

This review has some limitations; the chief among them is the inadequacy of participants. Available studies for each outcome fail to provide a sufficient sample size of more than 5,000 totally, which was needed to produce a definitive conclusion. As most of the participants in this study were elderly people over 50 years old and male patients accounted for the majority, the conclusions obtained in this review were more applicable to elderly male patients with CHD. However, due to the age and gender tendency of CHD, we did not use age or gender ratio as a special factor to perform a subgroup analysis and bias evaluation. It was hard to explain inconsistent conclusions on total plaque volume and volume of the main plaque compositions. The weak robustness of some of the outcomes needs to be noted. There have been noticeable changes in both the result and heterogeneity of some outcomes when some studies were excluded. Hence, some conclusions may be considered more cautiously. We fail to determine the specific linear relationship between the change of total atherosclerotic plaques volume and volume of the plaque compositions because the volume change of plaque compositions cannot be judged under the current statistical standard, which may need further studies. It is worth noting that a large portion of the high-quality studies interested in the effect of ω-3 PUFAs on inflammatory response focused on healthy individuals. This prevents us from directly demonstrating the relationship between ω-3 PUFAs and coronary inflammations.

Important questions remain regarding ω-3 PUFAs and coronary atherosclerosis. Chief among them is the lack of large-scale random controlled trials or cohort studies reporting the association between ω-3 PUFAs and coronary atherosclerosis. Currently available studies only include dozens to more than 100 samples, which was thought to be unable to produce more convincing conclusions. The portion of the studies included in this review had a short follow-up period, which is also a common problem in many current clinical studies on ω-3 PUFAs. More studies with long-term follow-up are needed to confirm the effects of ω-3 PUFAs on the cardiovascular system through long-term effects on lipids or inflammatory cytokine levels. Larger-scale studies are needed to confirm the effect of ω-3 PUFAs so as to provide more credible evidence.

We confirmed that ω-3 PUFAs benefit patients with CHD by reducing the progression of coronary atherosclerosis. We indicated that the benefits may not be caused by reducing endothelial inflammations of coronary arteries.

## Data Availability Statement

The original contributions presented in the study are included in the article/Supplementary Material, further inquiries can be directed to the corresponding author.

## Author Contributions

ZG, DZ, XX, and XY contributed to the literature search, study design and identification, and data acquisition and recording of the characteristics of studies. ZG contributed to the data analysis. DZ contributed to the data review and correction. ZG, DZ, HS, XX, and XY contributed to the data interpretation and critical revision of the manuscript. HS embellished the images and further interpreted the data. All authors contributed to the article and approved the submitted version.

## Conflict of Interest

The authors declare that the research was conducted in the absence of any commercial or financial relationships that could be construed as a potential conflict of interest.

## Publisher's Note

All claims expressed in this article are solely those of the authors and do not necessarily represent those of their affiliated organizations, or those of the publisher, the editors and the reviewers. Any product that may be evaluated in this article, or claim that may be made by its manufacturer, is not guaranteed or endorsed by the publisher.

## References

[1] Writing Group for the AREDS2 Research Group Bonds DE Harrington M Worrall BB Bertoni AG Eaton CB. Effect of long-chain ω-3 fatty acids and lutein + zeaxanthin supplements on cardiovascular outcomes: results of the Age-Related Eye Disease Study 2 (AREDS2) randomized clinical trial. JAMA Intern Med. (2014) 174:763–71. 10.1001/jamainternmed.2014.328 24638908

[2] Marchioli R Marfisi RM Borrelli G Chieffo C Franzosi MG Levantesi G . Efficacy of n-3 polyunsaturated fatty acids according to clinical characteristics of patients with recent myocardial infarction: insights from the GISSI-Prevenzione trial. J Cardiovasc Med. (2007) 8(Suppl. 1):S34–7. 10.2459/01.JCM.0000289271.80180.b6 17876196

[3] Skulas-Ray AC Wilson PWF Harris WS Brinton EA Kris-Etherton PM Richter CK . Omega-3 fatty acids for the management of hypertriglyceridemia: a science advisory from the American Heart Association. Circulation. (2019) 140:e673–91. 10.1161/CIR.0000000000000709 31422671

[4] Rudkowska I Caron-Dorval D Verreault M Couture P Deshaies Y Barbier O . PPARalpha L162V polymorphism alters the potential of n-3 fatty acids to increase lipoprotein lipase activity. Mol Nutr Food Res. (2010) 54:543–50. 10.1002/mnfr.200900085 19937854

[5] Calder PC. Omega-3 fatty acids and inflammatory processes: from molecules to man. Biochem Soc Trans. (2017) 45:1105–15. 10.1042/BST20160474 28900017

[6] Harris WS Rambjør GS Windsor SL Diederich D. n-3 fatty acids and urinary excretion of nitric oxide metabolites in humans. Am J Clin Nutr. (1997) 65:459–64. 10.1093/ajcn/65.2.459 9022531

[7] Goua M Mulgrew S Frank J Rees D Sneddon AA Wahle KW. Regulation of adhesion molecule expression in human endothelial and smooth muscle cells by omega-3 fatty acids and conjugated linoleic acids: involvement of the transcription factor NF-kappaB. Prostaglandins Leukot Essent Fatty Acids. (2008) 78:33–43. 10.1016/j.plefa.2007.10.004 18036803

[8] Preston Mason R. New insights into mechanisms of action for omega-3 fatty acids in atherothrombotic cardiovascular disease. Curr Atheroscler Rep. (2019) 21:2. 10.1007/s11883-019-0762-1 30637567 PMC6330561

[9] Kienzler JL Gold M Nollevaux F. Systemic bioavailability of topical diclofenac sodium gel 1% versus oral diclofenac sodium in healthy volunteers. J Clin Pharmacol. (2010) 50:50–61. 10.1177/0091270009336234 19841157

[10] Kaur G Cameron-Smith D Garg M Sinclair AJ. Docosapentaenoic acid (22:5n-3): a review of its biological effects. Prog Lipid Res. (2011) 50:28–34. 10.1016/j.plipres.2010.07.004 20655949

[11] Sakamoto A Saotome M Iguchi K Maekawa Y. Marine-derived omega-3 polyunsaturated fatty acids and heart failure: current understanding for basic to clinical relevance. Int J Mol Sci. (2019) 20:4025. 10.3390/ijms20164025 31426560 PMC6719114

[12] Banach M Serban C Sahebkar A Mikhailidis DP Ursoniu S Ray KK . Impact of statin therapy on coronary plaque composition: a systematic review and meta-analysis of virtual histology intravascular ultrasound studies. BMC Med. (2015) 13:229. 10.1186/s12916-015-0459-4 26385210 PMC4575433

[13] Vergallo R Crea F. Atherosclerotic plaque healing. N Engl J Med. (2020) 383:846–57. 10.1056/NEJMra2000317 32846063

[14] Balk EM Lichtenstein AH Chung M Kupelnick B Chew P Lau J. Effects of omega-3 fatty acids on coronary restenosis, intima-media thickness, and exercise tolerance: a systematic review. Atherosclerosis. (2006) 184:237–46. 10.1016/j.atherosclerosis.2005.06.042 16084516

[15] Laake K Seljeflot I Fagerland MW Njerve IU Arnesen H Solheim S. Effects on serum fractalkine by diet and omega-3 fatty acid intervention: relation to clinical outcome. Mediators Inflamm. (2015) 2015:373070. 10.1155/2015/373070 25733777 PMC4334932

[16] Liberati A Altman DG Tetzlaff J Mulrow C Gøtzsche PC Ioannidis JP . The PRISMA statement for reporting systematic reviews and meta-analyses of studies that evaluate healthcare interventions: explanation and elaboration. BMJ. (2009) 339:b2700. 10.1136/bmj.b2700 19622552 PMC2714672

[17] Cumpston M Li T Page MJ Chandler J Welch VA Higgins JP . Updated guidance for trusted systematic reviews: a new edition of the Cochrane Handbook for Systematic Reviews of Interventions. Cochrane Database Syst Rev. (2019) 10:ED000142. 10.1002/14651858.ED000142 31643080 PMC10284251

[18] Siscovick DS Barringer TA Fretts AM Wu JH Lichtenstein AH Costello RB . Omega-3 polyunsaturated fatty acid (fish oil) supplementation and the prevention of clinical cardiovascular disease: a science advisory from the american heart association. Circulation. (2017) 135:e867–84. 28289069 10.1161/CIR.0000000000000482 PMC6903779

[19] Aucoin M Cooley K Knee C Fritz H Balneaves LG Breau R . Fish-derived omega-3 fatty acids and prostate cancer: a systematic review. Integr Cancer Ther. (2017) 16:32–62. 10.1177/1534735416656052 27365385 PMC5736071

[20] Yang K Zeng L Bao T Ge J. Effectiveness of omega-3 fatty acid for polycystic ovary syndrome: a systematic review and meta-analysis. Reprod Biol Endocrinol. (2018) 16:27. 10.1186/s12958-018-0346-x 29580250 PMC5870911

[21] Hu Y Hu FB Manson JE. Marine omega-3 supplementation and cardiovascular disease: an updated meta-analysis of 13 randomized controlled trials involving 127 477 participants. J Am Heart Assoc. (2019) 8:e013543. 10.1161/JAHA.119.013543 31567003 PMC6806028

[22] Lyu XZ Sun F Zhan SY. [Risk related to bias assessment: (4) revised cochrane risk of bias tool for cluster-randomized control trials (RoB2.0)]. Zhonghua Liu Xing Bing Xue Za Zhi. (2018) 39:240–4. 10.3760/cma.j.issn.0254-6450.2018.02.020 29495213

[23] Higgins JP Thompson SG Deeks JJ Altman DG. Measuring inconsistency in meta-analyses. BMJ. (2003) 327:557–60. 10.1136/bmj.327.7414.557 12958120 PMC192859

[24] Alfaddagh A Elajami TK Ashfaque H Saleh M Bistrian BR Welty FK. Effect of Eicosapentaenoic and docosahexaenoic acids added to statin therapy on coronary artery plaque in patients with coronary artery disease: a randomized clinical trial. J Am Heart Assoc. (2017) 6:e006981. 10.1161/JAHA.117.006981 29246960 PMC5779017

[25] Ahn J Park SK Park TS Kim JH Yun E Kim SP . Effect of n-3 polyunsaturated fatty acids on regression of coronary atherosclerosis in statin treated patients undergoing percutaneous coronary intervention. Korean Circ J. (2016) 46:481–9. 10.4070/kcj.2016.46.4.481 27482256 PMC4965426

[26] Lee KW Blann AD Lip GY. Effects of omega-3 polyunsaturated fatty acids on plasma indices of thrombogenesis and inflammation in patients post-myocardial infarction. Thromb Res. (2006) 118:305–12. 10.1016/j.thromres.2005.07.018 16154181

[27] Gillingham LG Gustafson JA Han SY Jassal DS Jones PJ. High-oleic rapeseed (canola) and flaxseed oils modulate serum lipids and inflammatory biomarkers in hypercholesterolaemic subjects. Br J Nutr. (2011) 105:417–27. 10.1017/S0007114510003697 20875216

[28] Phang M Scorgie FE Seldon M Garg ML Lincz LF. Reduction of prothrombin and Factor V levels following supplementation with omega-3 fatty acids is sex dependent: a randomised controlled study. J Nutr Biochem. (2014) 25:997–1002. 10.1016/j.jnutbio.2014.05.001 24997005

[29] Mirfatahi M Tabibi H Nasrollahi A Hedayati M Taghizadeh M. Effect of flaxseed oil on serum systemic and vascular inflammation markers and oxidative stress in hemodialysis patients:a randomized controlled trial. Int Urol Nephrol. (2016) 48:1335–41. 10.1007/s11255-016-1300-5 27115157

[30] Berstad P Seljeflot I Veierød MB Hjerkinn EM Arnesen H Pedersen JI. Supplementation with fish oil affects the association between very long-chain n-3 polyunsaturated fatty acids in serum non-esterified fatty acids and soluble vascular cell adhesion molecule-1. Clin Sci. (2003) 105:13–20. 10.1042/CS20020349 12589702

[31] Wu SY Mayneris-Perxachs J Lovegrove JA Todd S Yaqoob P. Fish-oil supplementation alters numbers of circulating endothelial progenitor cells and microparticles independently of eNOS genotype. Am J Clin Nutr. (2014) 100:1232–43. 10.3945/ajcn.114.088880 25332321

[32] Amano T Matsubara T Uetani T Kato M Kato B Yoshida T . Impact of omega-3 polyunsaturated fatty acids on coronary plaque instability: an integrated backscatter intravascular ultrasound study. Atherosclerosis. (2011) 218:110–6. 10.1016/j.atherosclerosis.2011.05.030 21684546

[33] Kita Y Watanabe M Kamon D Ueda T Soeda T Okayama S . Effects of fatty acid therapy in addition to strong statin on coronary plaques in acute coronary syndrome: an optical coherence tomography study. J Am Heart Assoc. (2020) 9:e015593. 10.1161/JAHA.119.015593 32805184 PMC7660823

[34] Sugizaki Y Otake H Kuroda K Kawamori H Toba T Nagasawa A . Concomitant use of rosuvastatin and eicosapentaenoic acid significantly prevents native coronary atherosclerotic progression in patients with in-stent neoatherosclerosis. Circ J. (2020) 84:1826–36. 10.1253/circj.CJ-20-0199 32759543

[35] Alfaddagh A Elajami TK Saleh M Mohebali D Bistrian BR Welty FK. An omega-3 fatty acid plasma index ≥4% prevents progression of coronary artery plaque in patients with coronary artery disease on statin treatment. Atherosclerosis. (2019) 285:153–62. 10.1016/j.atherosclerosis.2019.04.213 31055222 PMC7963401

[36] Hjerkinn EM Seljeflot I Ellingsen I Berstad P Hjermann I Sandvik L . Influence of long-term intervention with dietary counseling, long-chain n-3 fatty acid supplements, or both on circulating markers of endothelial activation in men with long-standing hyperlipidemia. Am J Clin Nutr. (2005) 81:583–9. 10.1093/ajcn/81.3.583 15755826

[37] Sacks FM Stone PH Gibson CM Silverman DI Rosner B Pasternak RC. Controlled trial of fish oil for regression of human coronary atherosclerosis. HARP Research Group. J Am Coll Cardiol. (1995) 25:1492–8. 10.1016/0735-1097(95)00095-L 7759696

[38] Tokgozoglu L Catapano AL. Can EPA evaporate plaques. Eur Heart J. (2020) 41:3933–5. 10.1093/eurheartj/ehaa750 33141163

[39] Johansen O Seljeflot I Høstmark AT Arnesen H. The effect of supplementation with omega-3 fatty acids on soluble markers of endothelial function in patients with coronary heart disease. Arterioscler Thromb Vasc Biol. (1999) 19:1681–6. 10.1161/01.ATV.19.7.1681 10397685

[40] Seierstad SL Seljeflot I Johansen O Hansen R Haugen M Rosenlund G . Dietary intake of differently fed salmon; the influence on markers of human atherosclerosis. Eur J Clin Invest. (2005) 35:52–9. 10.1111/j.1365-2362.2005.01443.x 15638820

[41] Watanabe T Ando K Daidoji H Otaki Y Sugawara S Matsui M . A randomized controlled trial of eicosapentaenoic acid in patients with coronary heart disease on statins. J Cardiol. (2017) 70:537–44. 10.1016/j.jjcc.2017.07.007 28863874

[42] von Schacky C Angerer P Kothny W Theisen K Mudra H. The effect of dietary omega-3 fatty acids on coronary atherosclerosis. A randomized, double-blind, placebo-controlled trial. Ann Intern Med. (1999) 130:554–62. 10.7326/0003-4819-130-7-199904060-00003 10189324

[43] Seljeflot I Arnesen H Brude IR Nenseter MS Drevon CA Hjermann I. Effects of omega-3 fatty acids and/or antioxidants on endothelial cell markers. Eur J Clin Invest. (1998) 28:629–35. 10.1046/j.1365-2362.1998.00336.x 9767357

[44] Niki T Wakatsuki T Yamaguchi K Taketani Y Oeduka H Kusunose K . Effects of the addition of eicosapentaenoic acid to strong statin therapy on inflammatory cytokines and coronary plaque components assessed by integrated backscatter intravascular ultrasound. Circ J. (2016) 80:450–60. 10.1253/circj.CJ-15-0813 26667367

[45] Erkkilä AT Matthan NR Herrington DM Lichtenstein AH. Higher plasma docosahexaenoic acid is associated with reduced progression of coronary atherosclerosis in women with CAD. J Lipid Res. (2006) 47:2814–9. 10.1194/jlr.P600005-JLR200 16983146

[46] Khan SU Lone AN Khan MS Virani SS Blumenthal RS Nasir K . Effect of omega-3 fatty acids on cardiovascular outcomes: a systematic review and meta-analysis. EClinicalMedicine. (2021) 38:100997. 10.1016/j.eclinm.2021.100997 34505026 PMC8413259

[47] Bucher HC Hengstler P Schindler C Meier G. N-3 polyunsaturated fatty acids in coronary heart disease: a meta-analysis of randomized controlled trials. Am J Med. (2002) 112:298–304. 10.1016/S0002-9343(01)01114-7 11893369

[48] Rizos EC Ntzani EE Bika E Kostapanos MS Elisaf MS. Association between omega-3 fatty acid supplementation and risk of major cardiovascular disease events: a systematic review and meta-analysis. JAMA. (2012) 308:1024–33. 10.1001/2012.jama.11374 22968891

[49] Kwak SM Myung SK Lee YJ Seo HG. Efficacy of omega-3 fatty acid supplements (eicosapentaenoic acid and docosahexaenoic acid) in the secondary prevention of cardiovascular disease: a meta-analysis of randomized, double-blind, placebo-controlled trials. Arch Intern Med. (2012) 172:686–94. 10.1001/archinternmed.2012.262 22493407

[50] Del Gobbo LC Imamura F Aslibekyan S Marklund M Virtanen JK Wennberg M . ω-3 Polyunsaturated fatty acid biomarkers and coronary heart disease: pooling project of 19 cohort studies. JAMA Intern Med. (2016) 176:1155–66. 10.1001/jamainternmed.2016.2925 27357102 PMC5183535

[51] Sharma G Martin SS Blumenthal RS. Effects of omega-3 fatty acids on major adverse cardiovascular events: what matters most: the drug, the dose, or the placebo. JAMA. (2020) 324:2262–4. 10.1001/jama.2020.22387 33190148

[52] Hino A Adachi H Toyomasu K Yoshida N Enomoto M Hiratsuka A . Very long chain N-3 fatty acids intake and carotid atherosclerosis: an epidemiological study evaluated by ultrasonography. Atherosclerosis. (2004) 176:145–9. 10.1016/j.atherosclerosis.2004.04.020 15306187

[53] Ebbesson SO Roman MJ Devereux RB Kaufman D Fabsitz RR Maccluer JW . Consumption of omega-3 fatty acids is not associated with a reduction in carotid atherosclerosis: the Genetics of Coronary Artery Disease in Alaska Natives study. Atherosclerosis. (2008) 199:346–53. 10.1016/j.atherosclerosis.2007.10.020 18054937

[54] Morisaki N Saito I Tamura K Tashiro J Masuda M Kanzaki T . New indices of ischemic heart disease and aging: studies on the serum levels of soluble intercellular adhesion molecule-1 (ICAM-1) and soluble vascular cell adhesion molecule-1 (VCAM-1) in patients with hypercholesterolemia and ischemic heart disease. Atherosclerosis. (1997) 131:43–8. 10.1016/S0021-9150(97)06083-8 9180243

[55] Hwang SJ Ballantyne CM Sharrett AR Smith LC Davis CE Gotto AM Jr . Circulating adhesion molecules VCAM-1, ICAM-1, and E-selectin in carotid atherosclerosis and incident coronary heart disease cases: the Atherosclerosis Risk In Communities (ARIC) study. Circulation. (1997) 96:4219–25. 10.1161/01.CIR.96.12.4219 9416885

[56] Kawakami A Aikawa M Alcaide P Luscinskas FW Libby P Sacks FM. Apolipoprotein CIII induces expression of vascular cell adhesion molecule-1 in vascular endothelial cells and increases adhesion of monocytic cells. Circulation. (2006) 114:681–7. 10.1161/CIRCULATIONAHA.106.622514 16894036

[57] Bossowska A Kiersnowska-Rogowska B Bossowski A Galar B Sowiński P. [Assessment of serum levels of adhesion molecules (sICAM-1, sVCAM-1, sE-selectin) in stable and unstable angina and acute myocardial infarction]. Przegl Lek. (2003) 60:445–50. 14750416

[58] Willeit P Thompson A Aspelund T Rumley A Eiriksdottir G Lowe G . Hemostatic factors and risk of coronary heart disease in general populations: new prospective study and updated meta-analyses. PLoS ONE. (2013) 8:e55175. 10.1371/journal.pone.0055175 23408959 PMC3567058

[59] van Schie MC de Maat MP Isaacs A van Duijn CM Deckers JW Dippel DW . Variation in the von Willebrand factor gene is associated with von Willebrand factor levels and with the risk for cardiovascular disease. Blood. (2011) 117:1393–9. 10.1182/blood-2010-03-273961 20940418

[60] Vischer UM. von Willebrand factor, endothelial dysfunction, and cardiovascular disease. J Thromb Haemost. (2006) 4:1186–93. 10.1111/j.1538-7836.2006.01949.x 16706957

[61] Innes JK Calder PC. The Differential effects of eicosapentaenoic acid and docosahexaenoic acid on cardiometabolic risk factors: a systematic review. Int J Mol Sci. (2018) 19:532. 10.3390/ijms19020532 29425187 PMC5855754

[62] Carracedo M Artiach G Arnardottir H Bäck M. The resolution of inflammation through omega-3 fatty acids in atherosclerosis, intimal hyperplasia, and vascular calcification. Semin Immunopathol. (2019) 41:757–66. 10.1007/s00281-019-00767-y 31696250 PMC6881483

[63] Bentzon JF Otsuka F Virmani R Falk E. Mechanisms of plaque formation and rupture. Circ Res. (2014) 114:1852–66. 10.1161/CIRCRESAHA.114.302721 24902970

[64] Ueda M. [Pathology of AtheroThrombosIS (ATIS)]. Drugs. (2010) 70(Suppl. 1):3–8. 10.2165/00000004-000000000-00000 20977286

[65] Jinnouchi H Sato Y Sakamoto A Cornelissen A Mori M Kawakami R . Calcium deposition within coronary atherosclerotic lesion: implications for plaque stability. Atherosclerosis. (2020) 306:85–95. 10.1016/j.atherosclerosis.2020.05.017 32654790

[66] Libby P Theroux P. Pathophysiology of coronary artery disease. Circulation. (2005) 111:3481–8. 10.1161/CIRCULATIONAHA.105.537878 15983262

[67] Vergallo R Porto I D'Amario D Annibali G Galli M Benenati S . Coronary atherosclerotic phenotype and plaque healing in patients with recurrent acute coronary syndromes compared with patients with long-term clinical stability: an *in vivo* optical coherence tomography study. JAMA Cardiol. (2019) 4:321–9. 10.1001/jamacardio.2019.0275 30865212 PMC6484796

[68] Aung T Halsey J Kromhout D Gerstein HC Marchioli R Tavazzi L . Associations of omega-3 fatty acid supplement use with cardiovascular disease risks: meta-analysis of 10 trials involving 77 917 individuals. JAMA Cardiol. (2018) 3:225–34. 10.1001/jamacardio.2017.5205 29387889 PMC5885893

[69] Abdelhamid AS Brown TJ Brainard JS Biswas P Thorpe GC Moore HJ . Omega-3 fatty acids for the primary and secondary prevention of cardiovascular disease. Cochrane Database Syst Rev. (2018) 7:CD003177. 10.1002/14651858.CD003177.pub4 30019766 PMC6513557

[70] Burillo E Martín-Fuentes P Mateo-Gallego R Baila-Rueda L Cenarro A Ros E . Omega-3 fatty acids and HDL. How do they work in the prevention of cardiovascular disease. Curr Vasc Pharmacol. (2012) 10:432–41. 10.2174/157016112800812845 22339302

[71] Mozaffarian D Wu JH. Omega-3 fatty acids and cardiovascular disease: effects on risk factors, molecular pathways, and clinical events. J Am Coll Cardiol. (2011) 58:2047–67. 10.1016/j.jacc.2011.06.063 22051327

