# Supplementary material for: Effects of ω-3 Polyunsaturated Fatty Acids on Coronary Atherosclerosis and Inflammation: A Systematic Review and Meta-Analysis
Source: Front Cardiovasc Med. 2022 Jun 20;9:904250. doi: 10.3389/fcvm.2022.904250 (PMC9251200; doi:10.3389/fcvm.2022.904250)
Supplement: Supplementary file 1 [file Data_Sheet_1.docx]

Supplementary Material

**
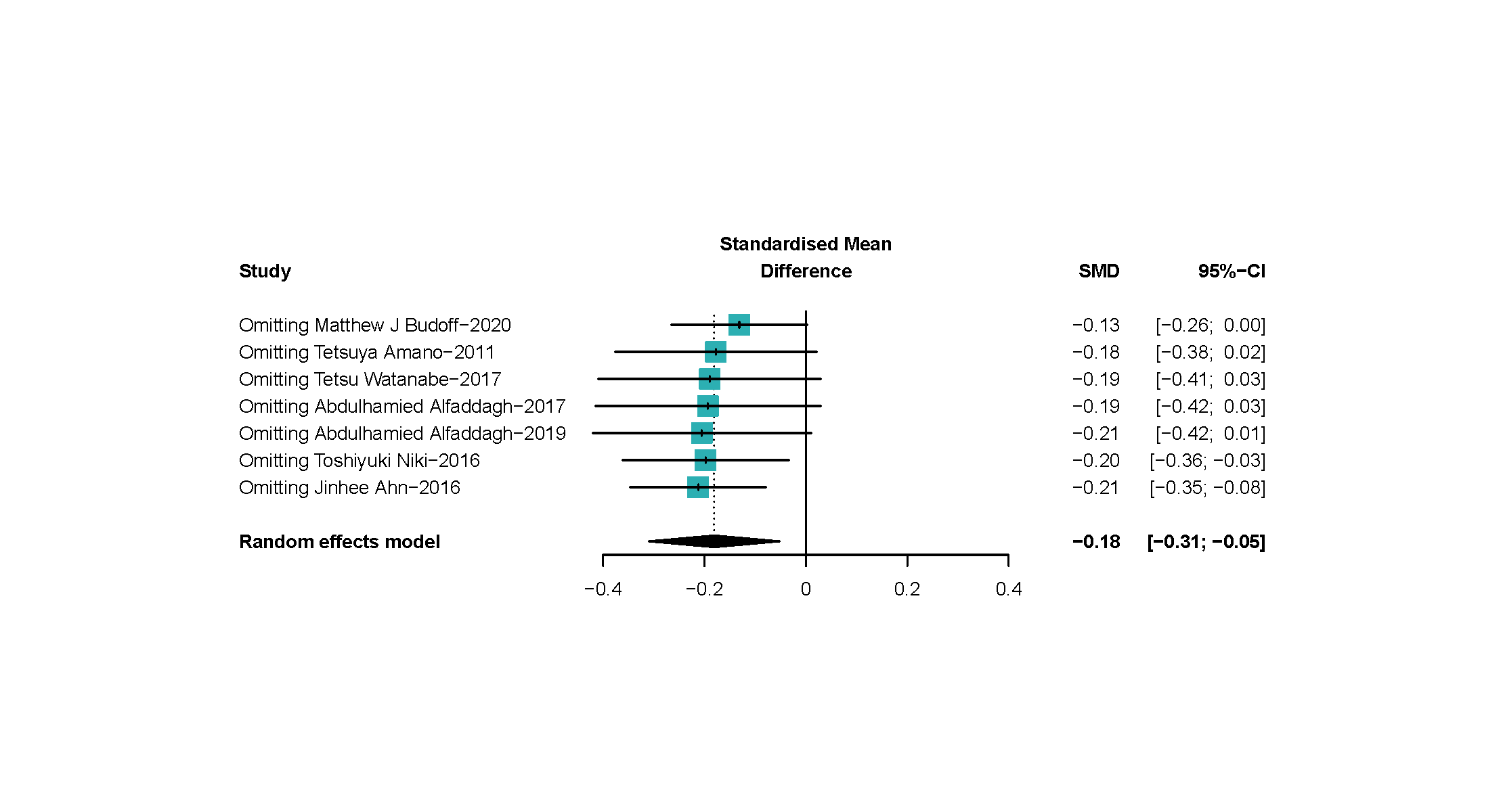
S Fig 1.1. Sensitivity analysis of ω-3 PUFAs supplementation and coronary atherosclerotic plaques. Omitting: study number. Data was calculated in a random-effects model.**

**
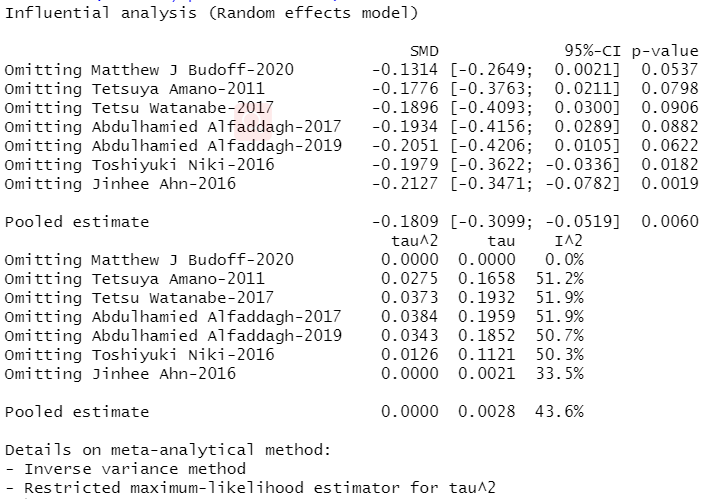
S Fig 1.2. Sensitivity analysis in R workstation of ω-3 PUFAs supplementation and coronary atherosclerotic plaques. Data was calculated in a random-effects model.**

**
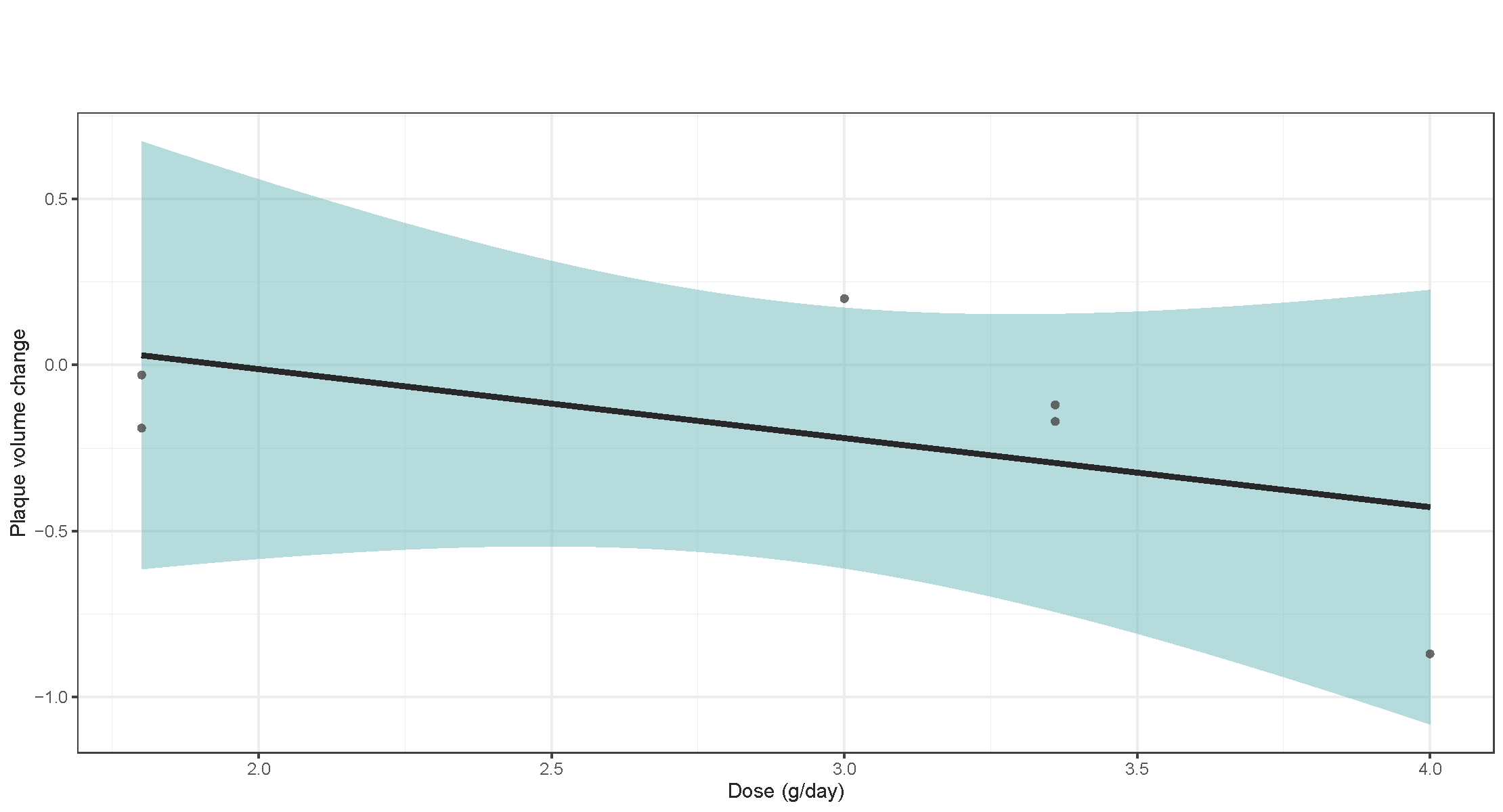
S Fig 1.3. Linear regression of ω-3 PUFAs supplementation dose and change in plaque volume**

**
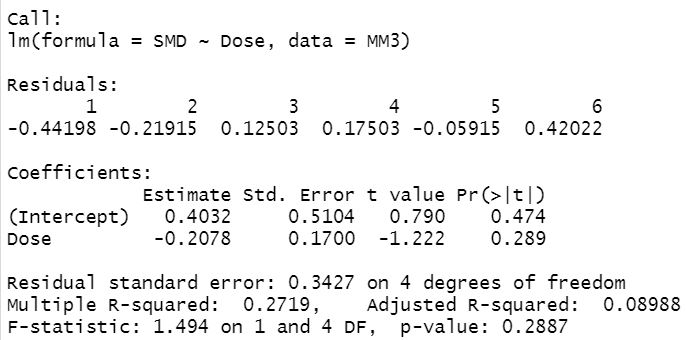
**

**S Fig 1.4. Linear regression results in R workstation of ω-3 PUFAs supplementation dose and change in plaque volume**

**
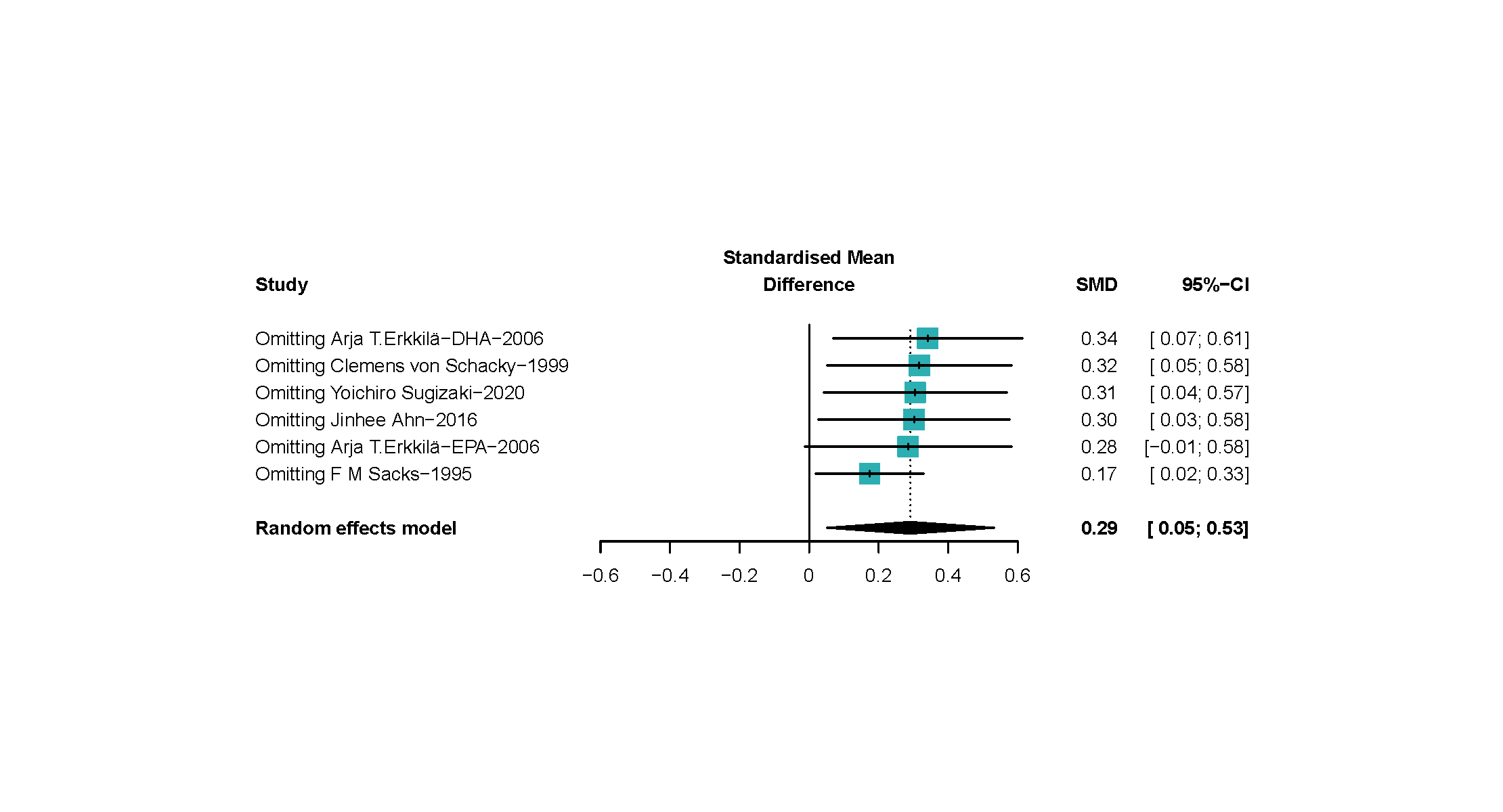
S Fig 2.1. Sensitivity analysis of associations of ω-3 PUFAs and most stenotic segment of the coronary arteries. Omitting: study number. Data was calculated in a random-effects model.**

**
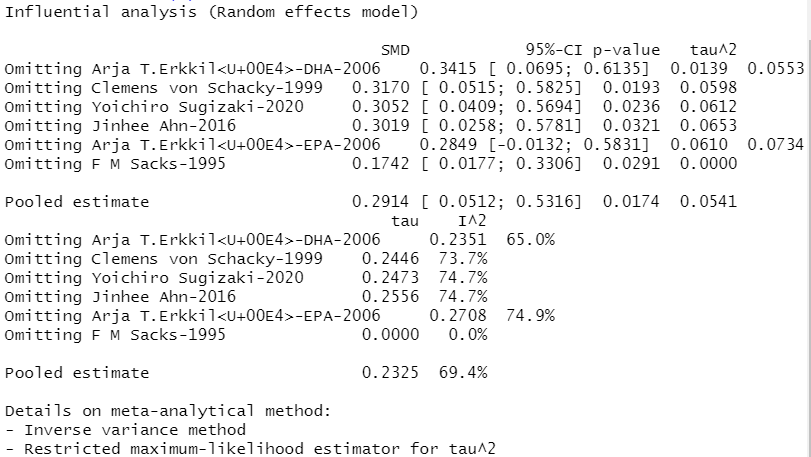
S Fig 2.2. Sensitivity analysis in R workstation of associations of ω-3 PUFAs and most stenotic segment of the coronary artery. Data was calculated in a random-effects model.**

**
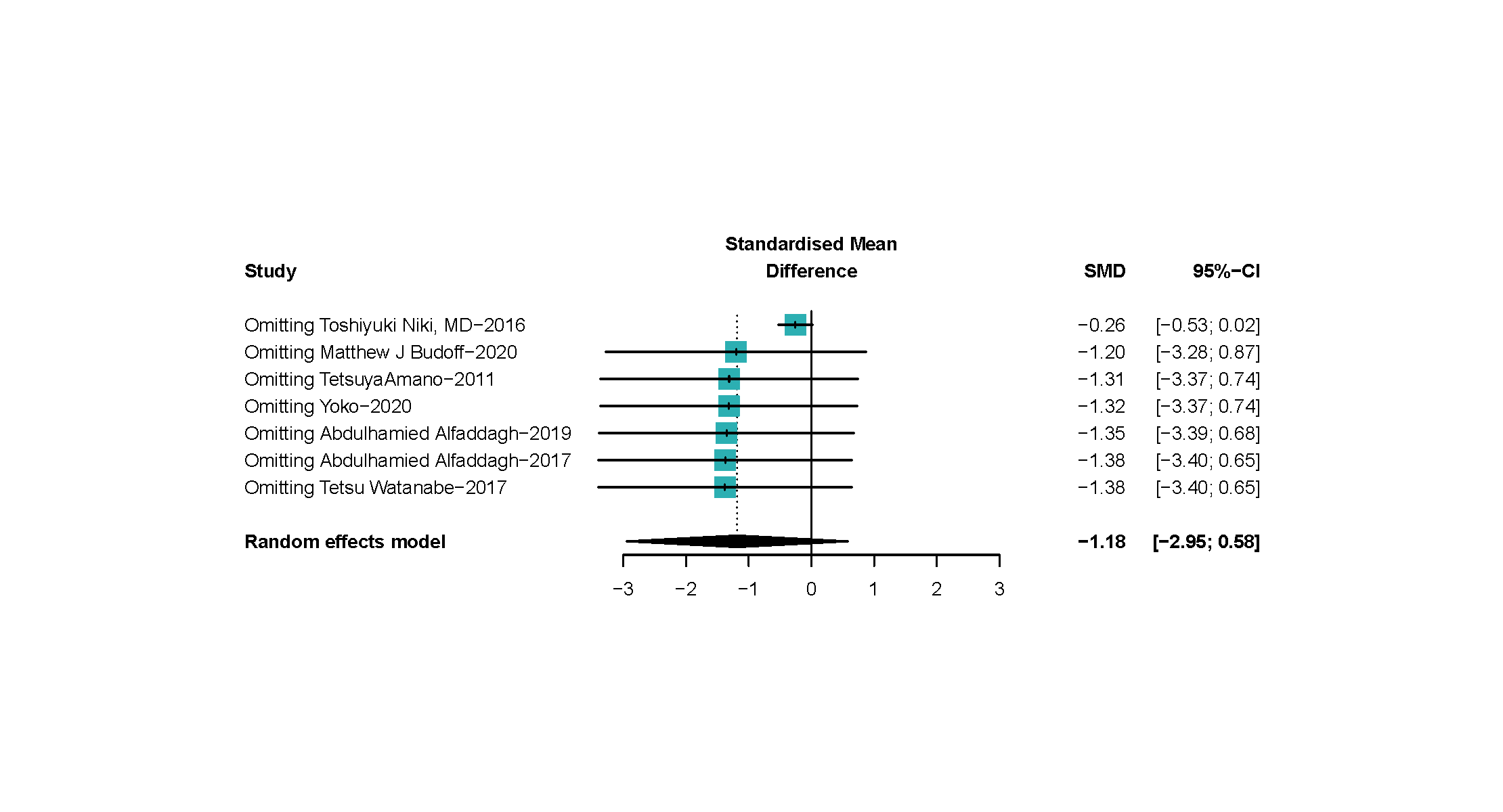
S Fig 3.1. Sensitivity analysis of ω-3 PUFAs and lipid plaques volume. Omitting: study number. Data was calculated in a random-effects model.**

**
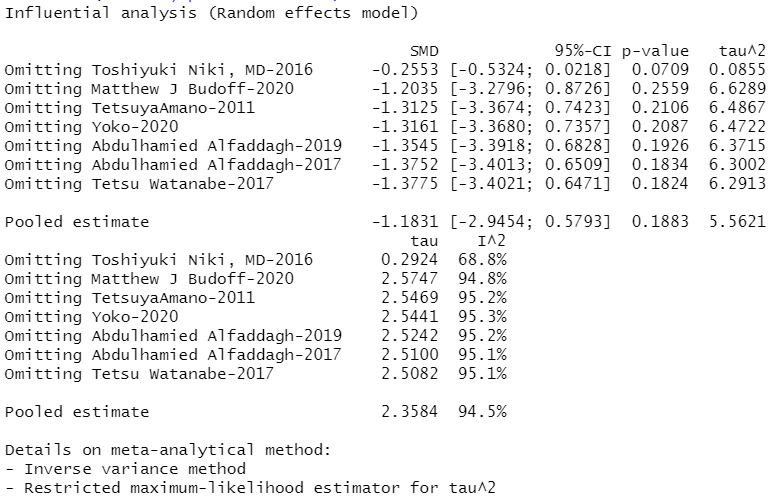
**

**S Fig 3.2. Sensitivity analysis in R workstation of ω-3 PUFAs ω-3 PUFAs and lipid plaques volume. Data was calculated in a random-effects model.**

**
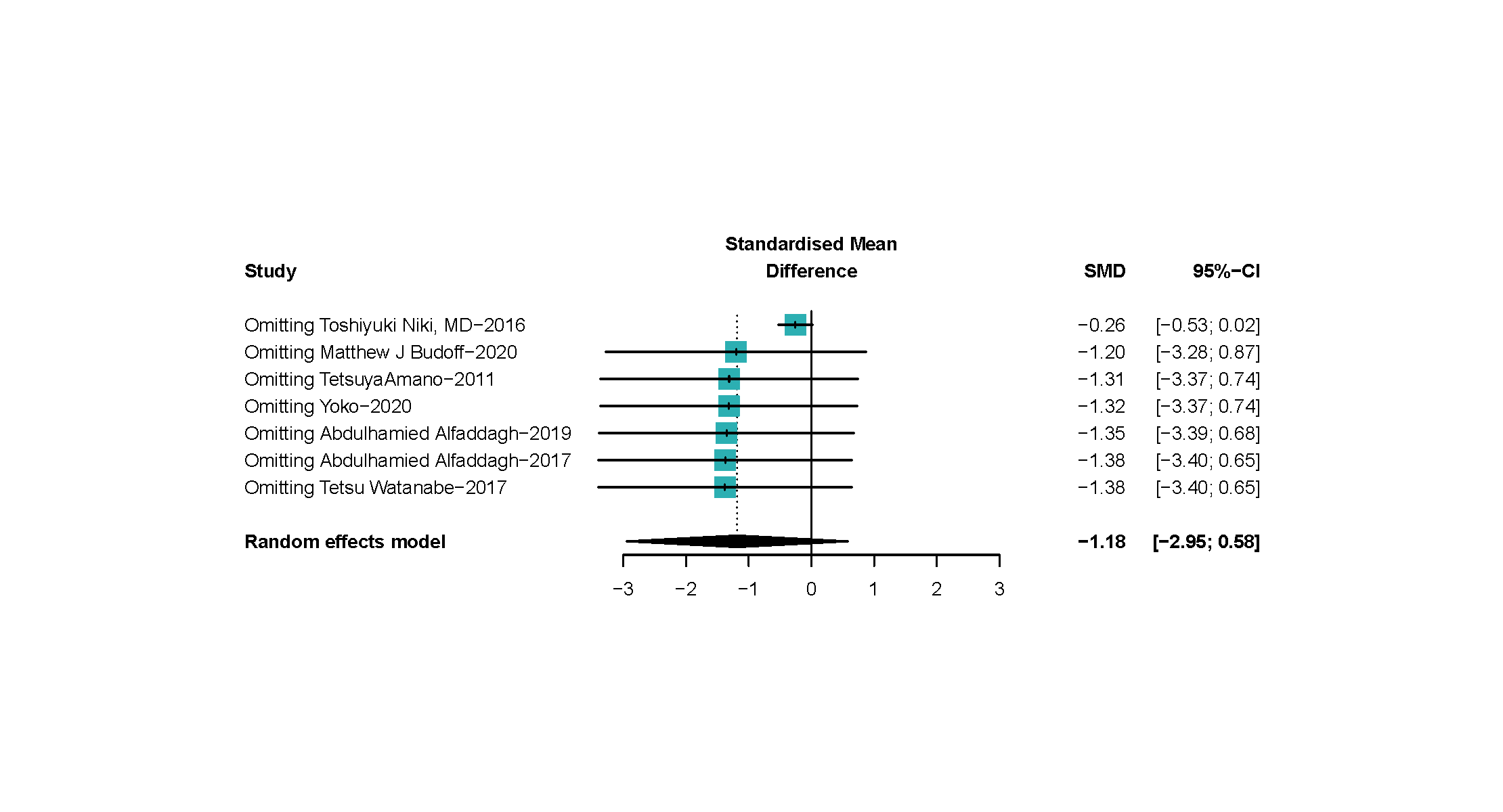
S Fig 4.1. Sensitivity analysis of ω-3 PUFAs and fiber plaque volume. Omitting: study number. Data was calculated in a random-effects model.**

**
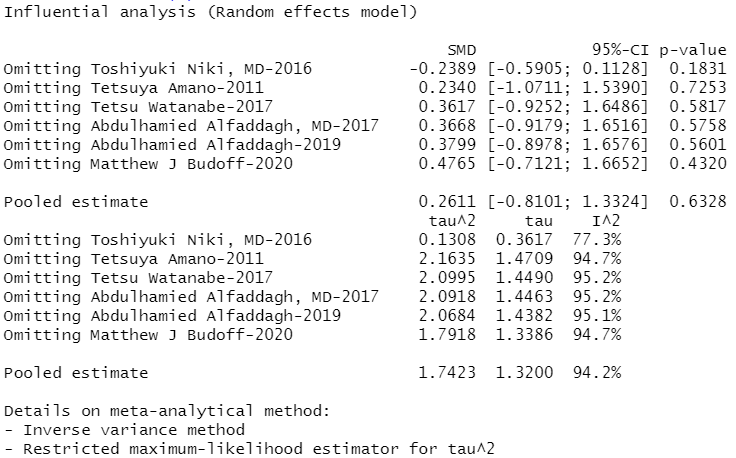
S Fig 4.2. Sensitivity analysis in R workstation of ω-3 PUFAs and coronary fiber plaque volume. Data was calculated in a random-effects model.**

**
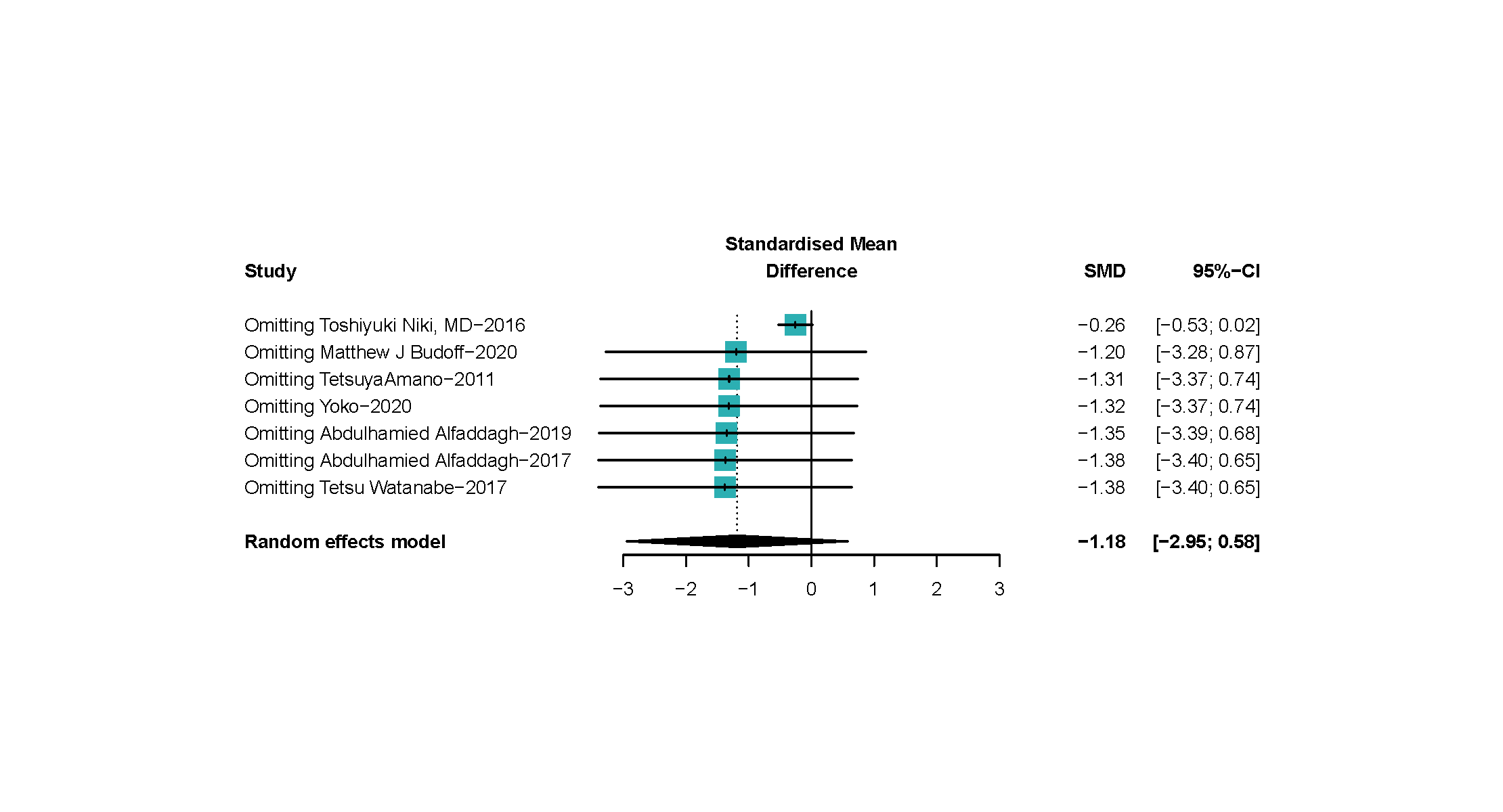
S Fig 5.1. Sensitivity analysis of ω-3 PUFAs and calcified plaque volume. Omitting: study number. Data was calculated in a random-effects model.**

**
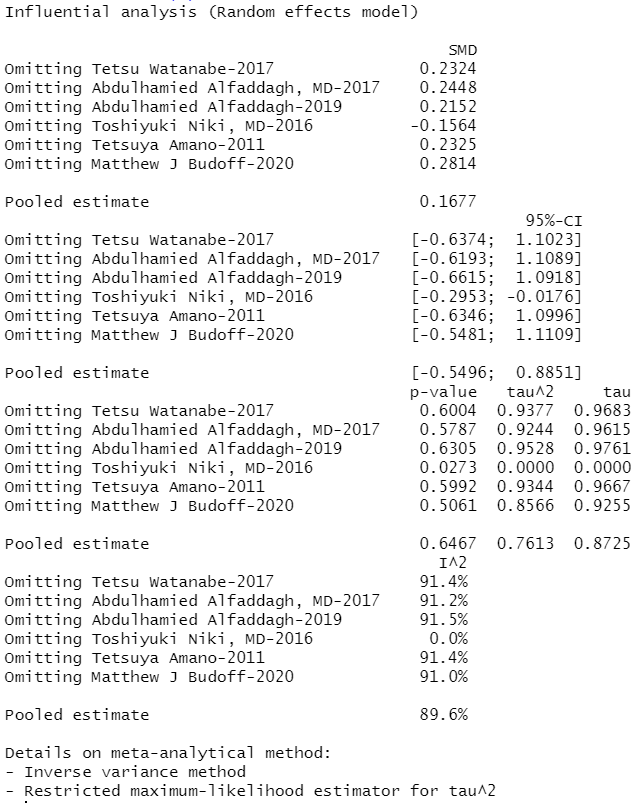
**

**S Fig 5.2. Sensitivity analysis in R workstation of ω-3 PUFAs and calcified plaque volume. Data was calculated in a random-effects model.**

**
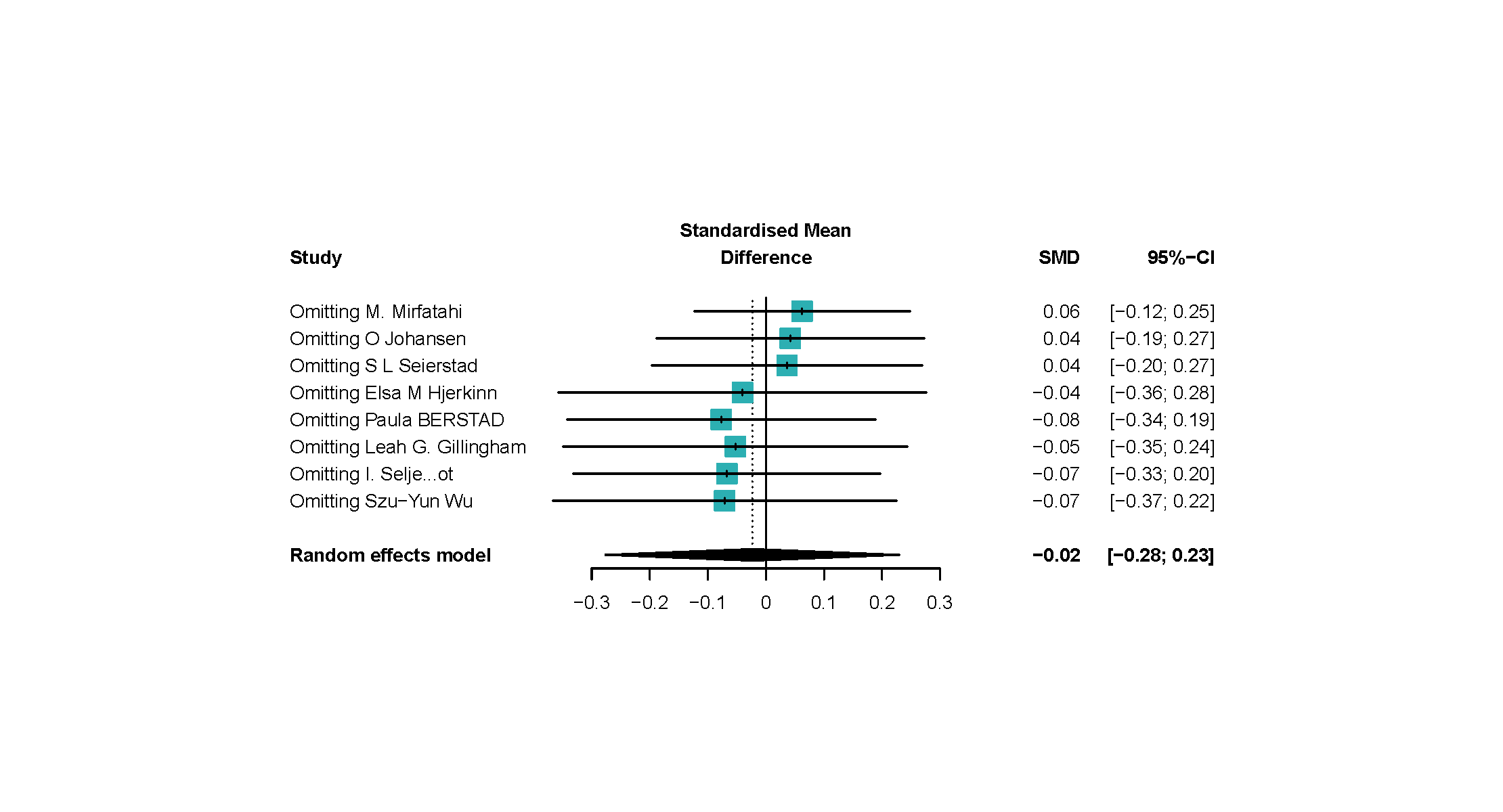
S Fig 6.1. Sensitivity analysis of ω-3 PUFAs and sVCAM-1 level. Omitting: study number. Data was calculated in a random-effects model.**

**
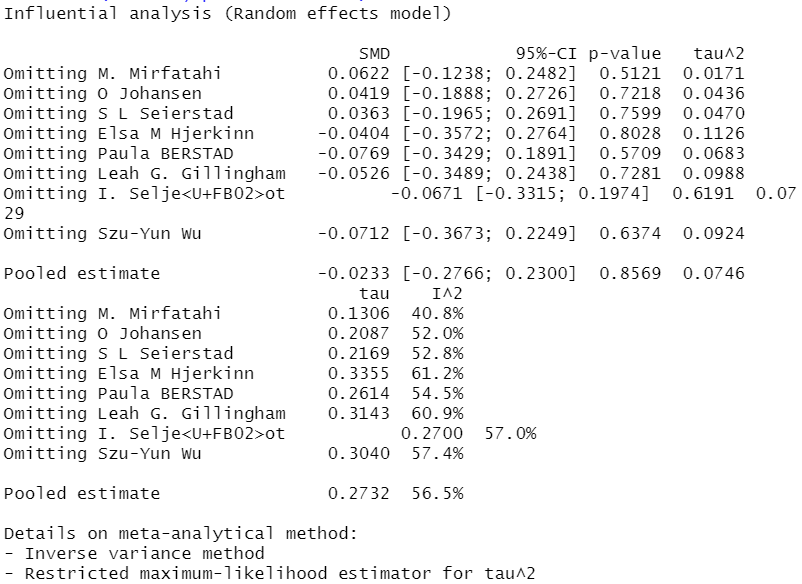
**

**S Fig 6.2. Sensitivity analysis in R workstation of ω-3 PUFAs and sVCAM-1 level. Data was calculated in a random-effects model.**

**
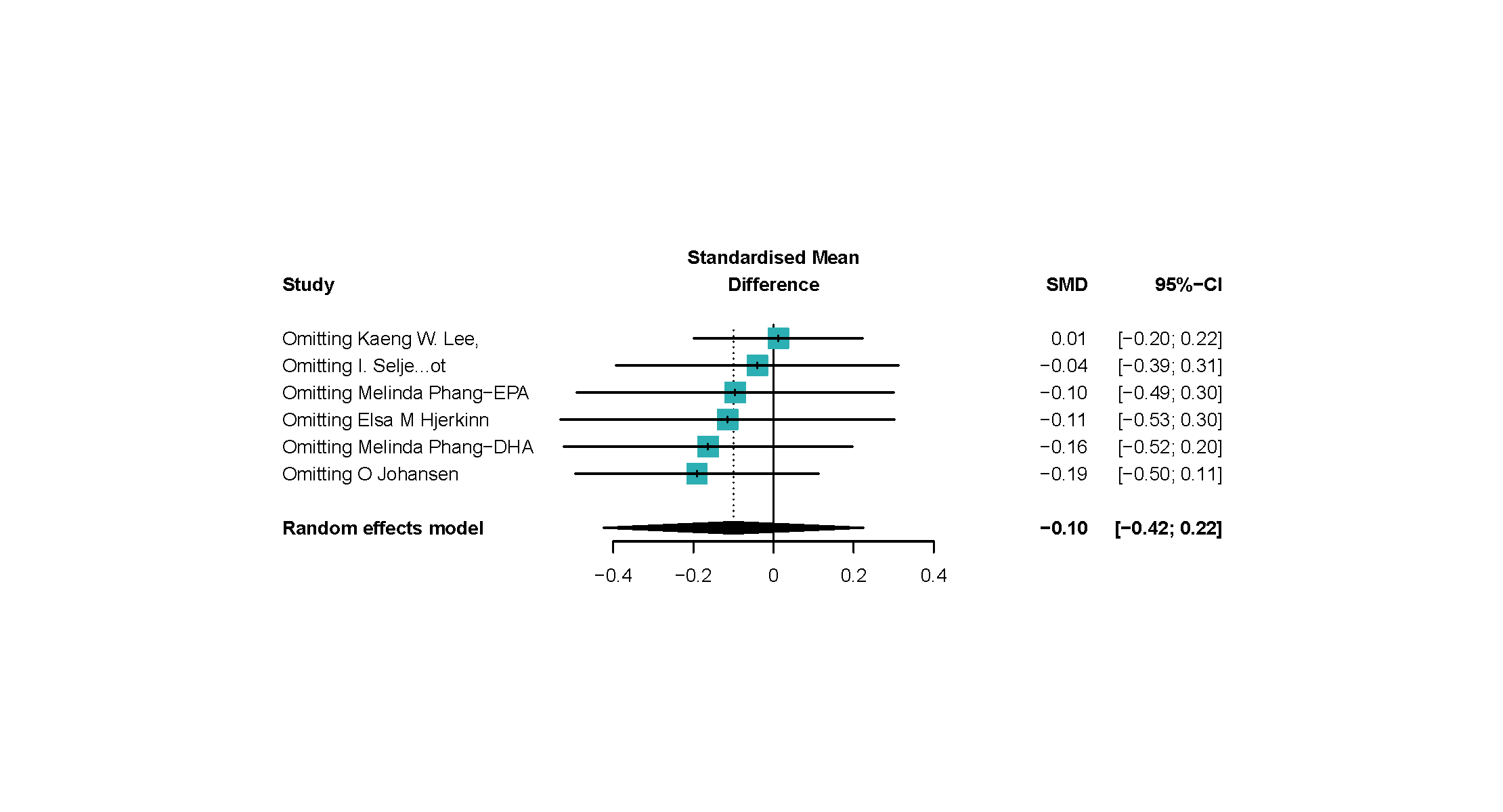
S Fig 7.1. Sensitivity analysis of ω-3 PUFAs and VWF%. Omitting: study number. Data was calculated in a random-effects model.**

**
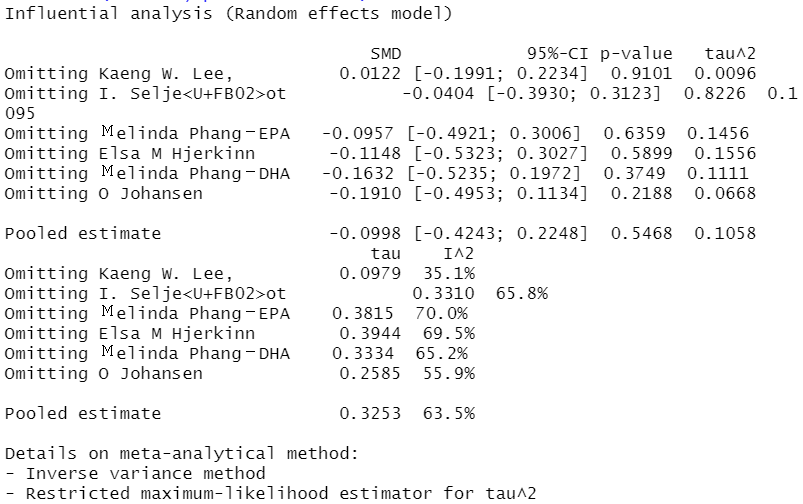
S Fig 7.2. Sensitivity analysis in R workstation of ω-3 PUFAs and VWF%. Data was calculated in a random-effects model.**

**
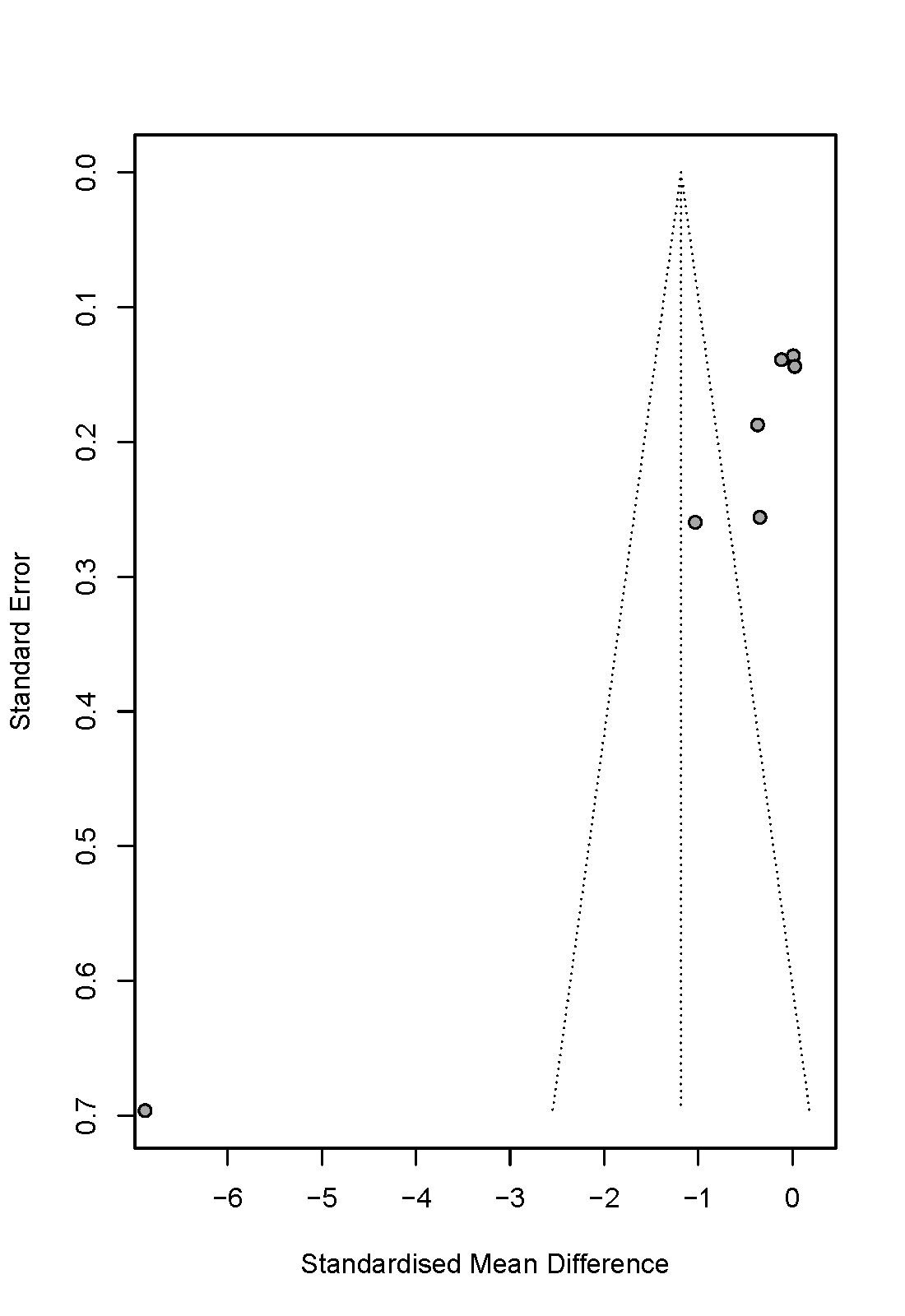
**

**S Fig 8.1 Funnel plot of ω-3 PUFAs and lipid plaques volume.**

**
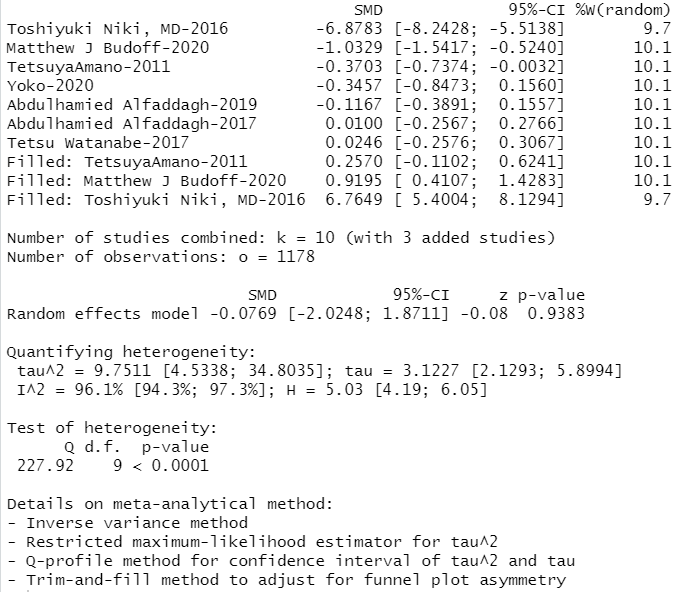
S Fig 8.2 R workstation of “trim and fill” of ω-3 PUFAs and lipid plaques volume.**

**
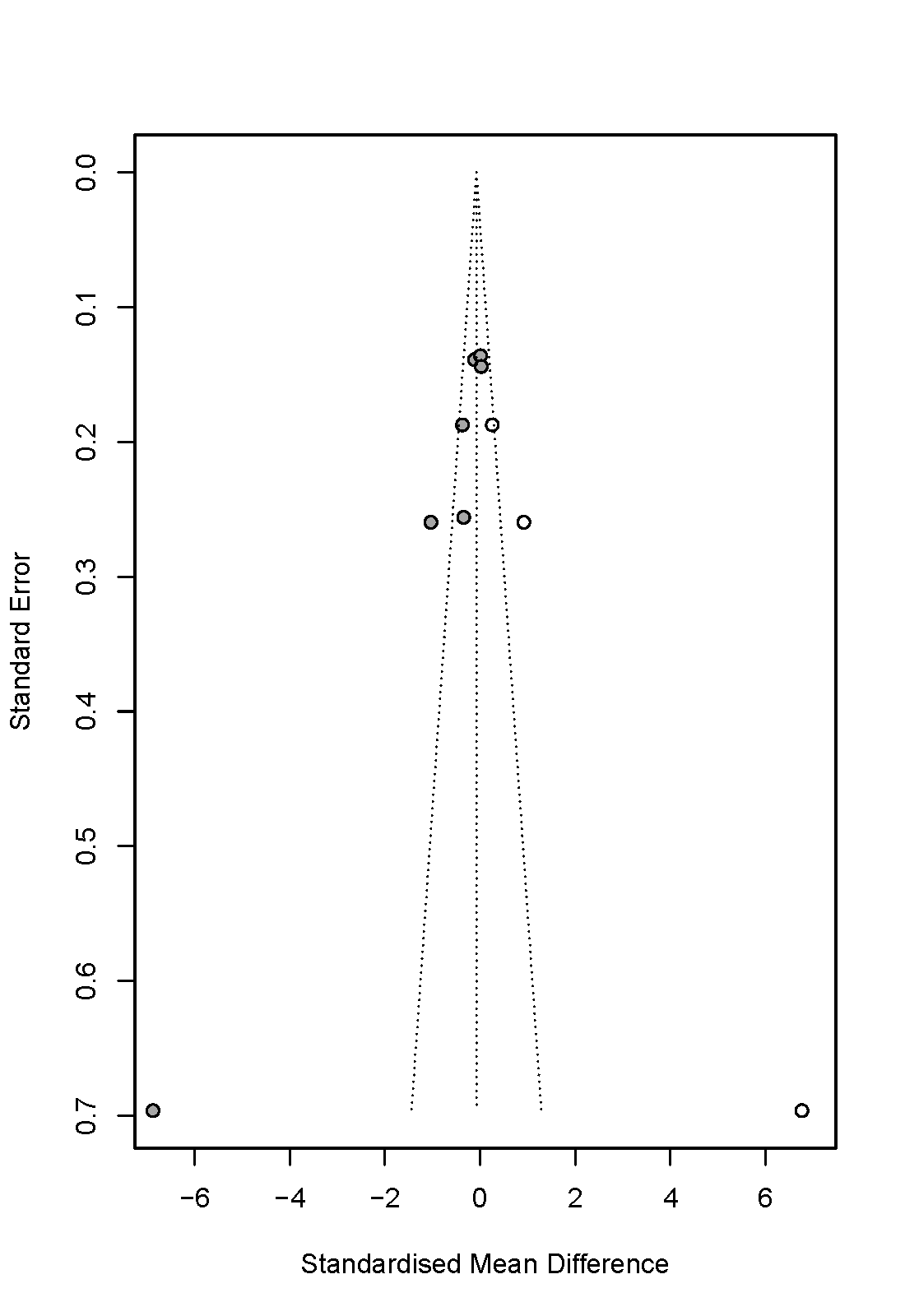
**

**S Fig 8.3 Trimmed funnel plot of ω-3 PUFAs and lipid plaques volume.**

**
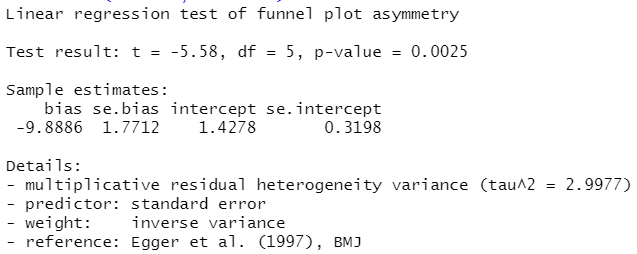
**

**S Fig 8.4 Peter’s test of ω-3 PUFAs and lipid plaques volume.**

**
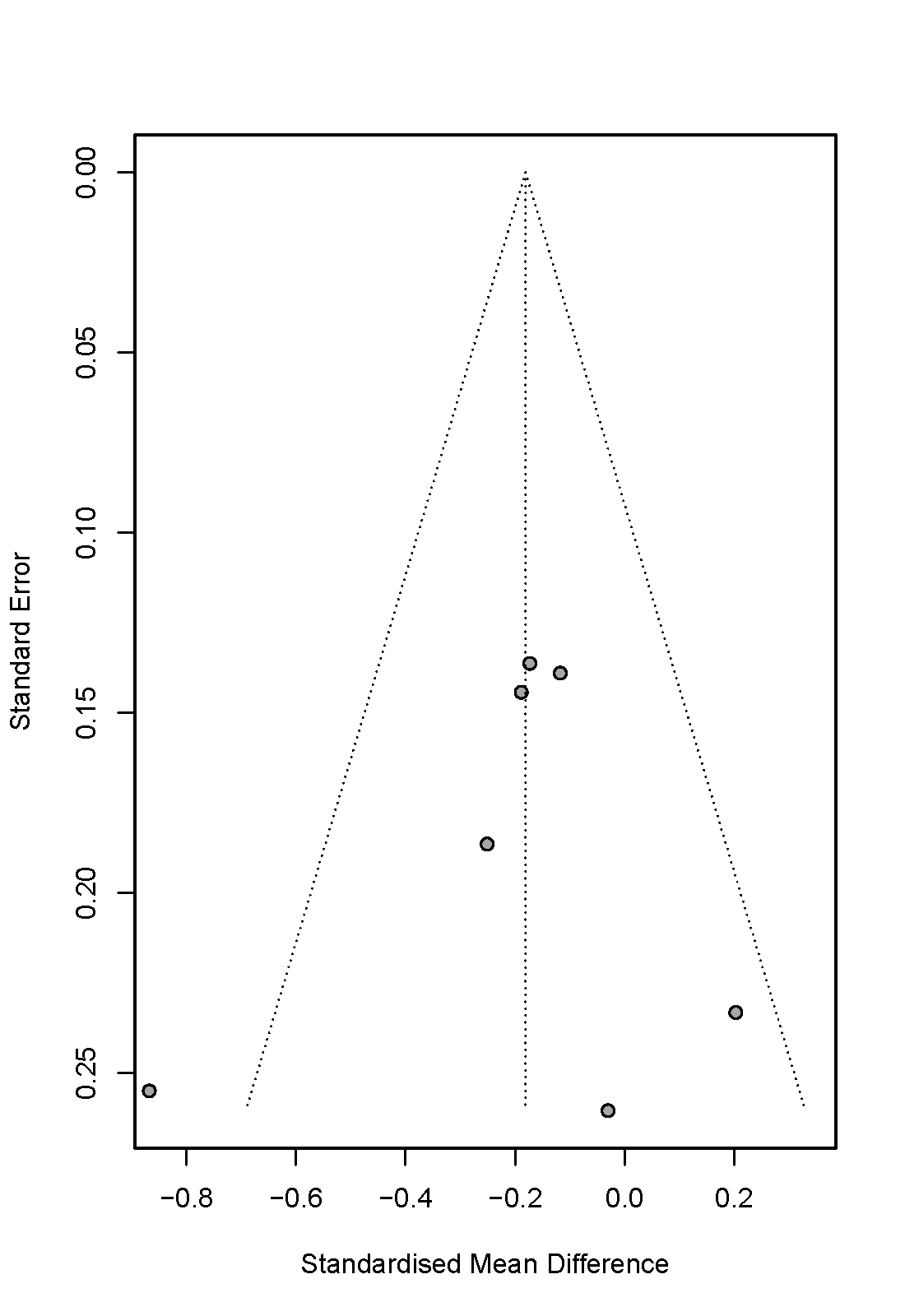
**

**S Fig 9.1 Funnel plot of ω-3 PUFAs supplementation and coronary atherosclerotic plaques.**

**
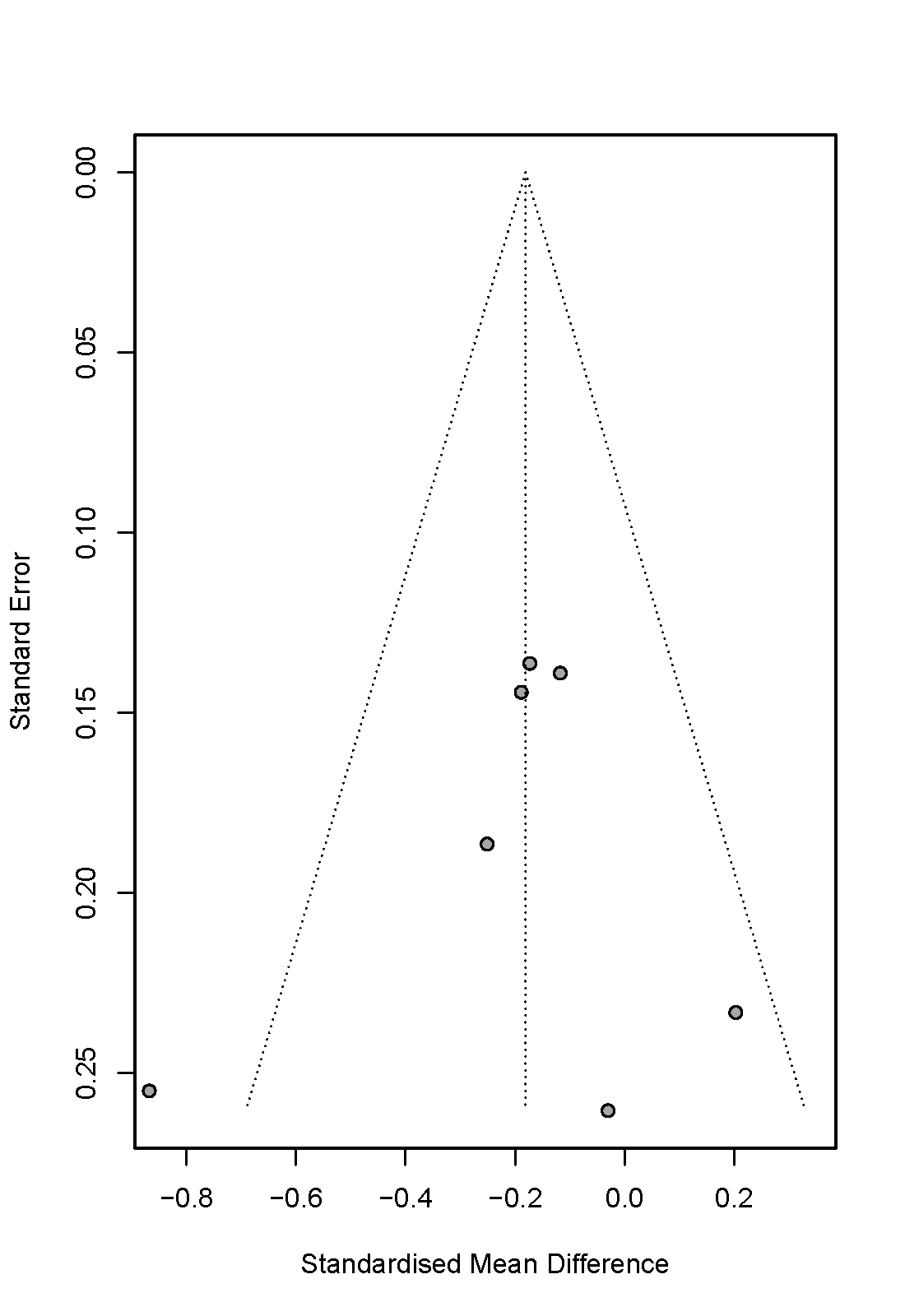
**

**S Fig 9.2 Funnel plot of ω-3 PUFAs and lipid plaques volume**

**
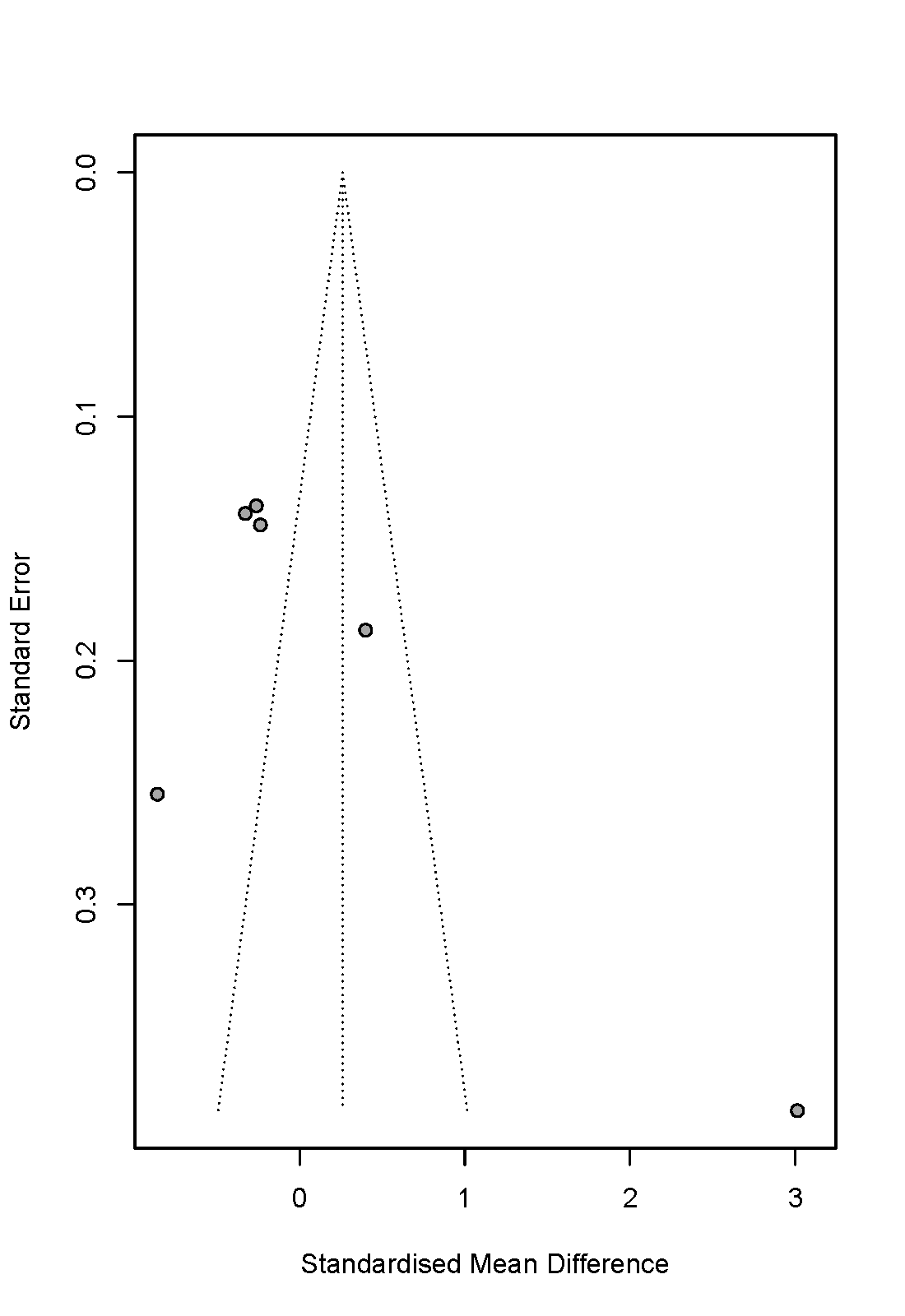
**

**S Fig 9.3 Funnel plot of ω-3 PUFAs and fiber plaques volume**

**
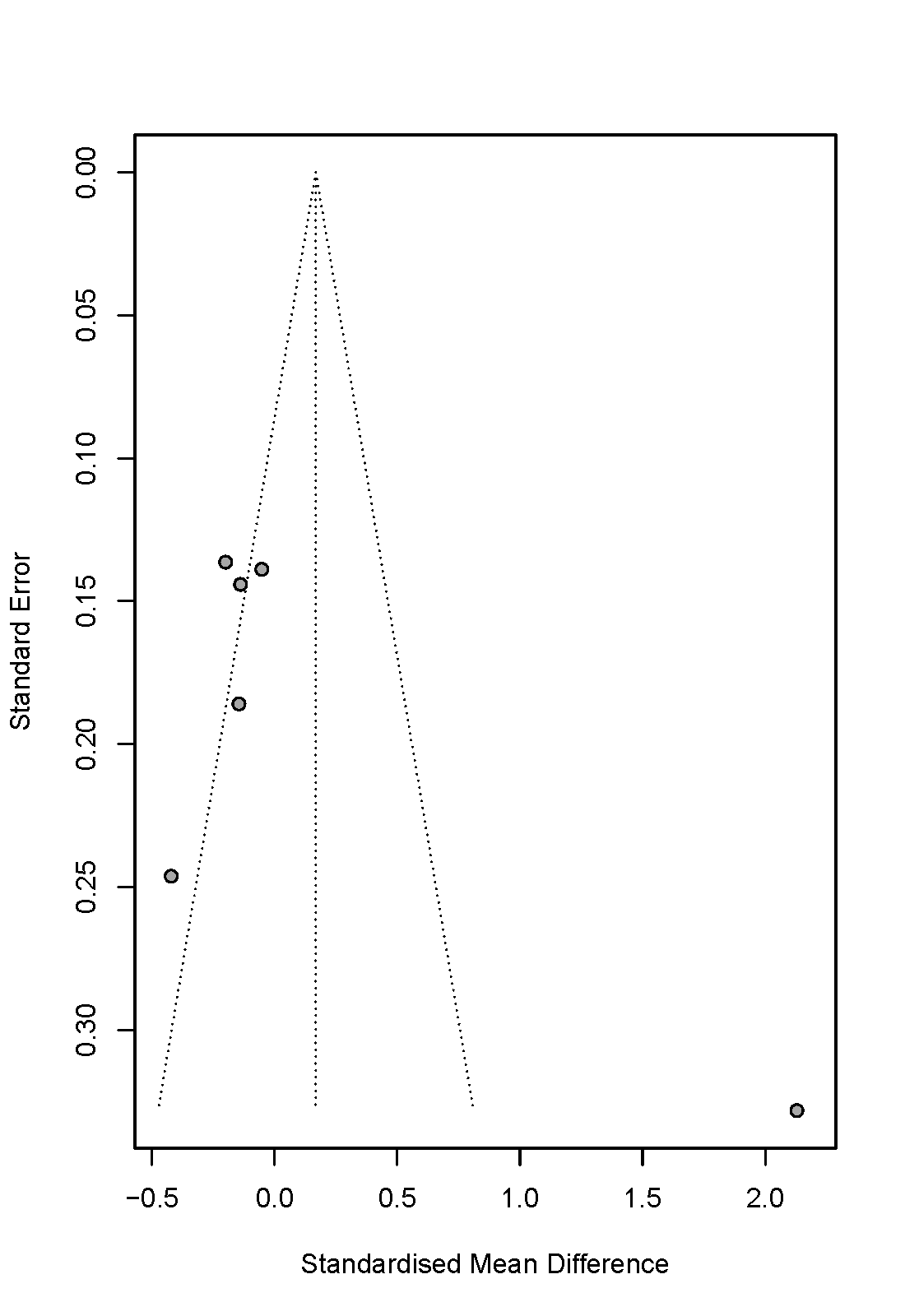
**

**S Fig 9.4 Funnel plot of ω-3 PUFAs and calcified plaque volume**

**
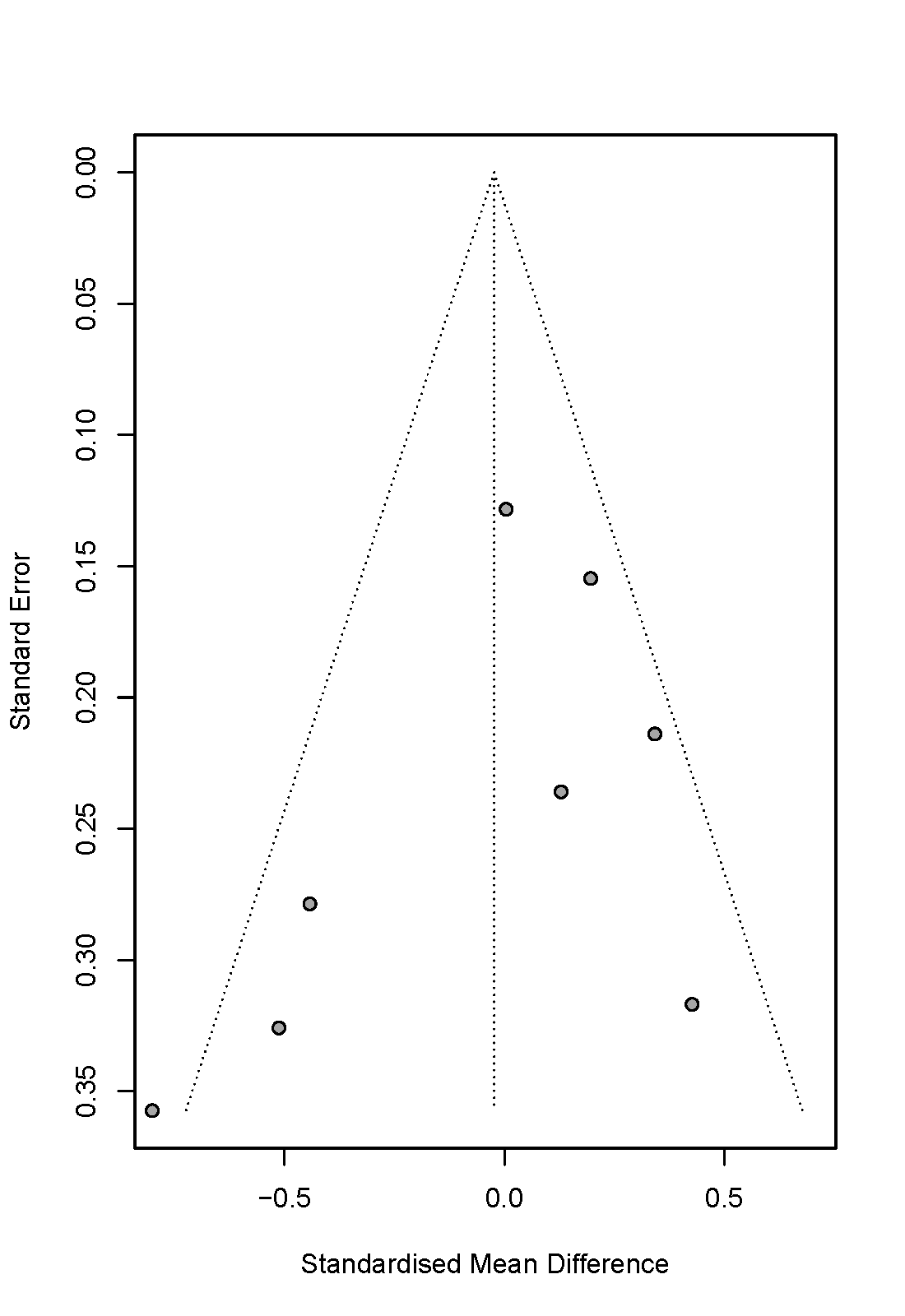
**

**S Fig 9.5 Funnel plot of ω-3 PUFAs and sVCAM-1.**

**
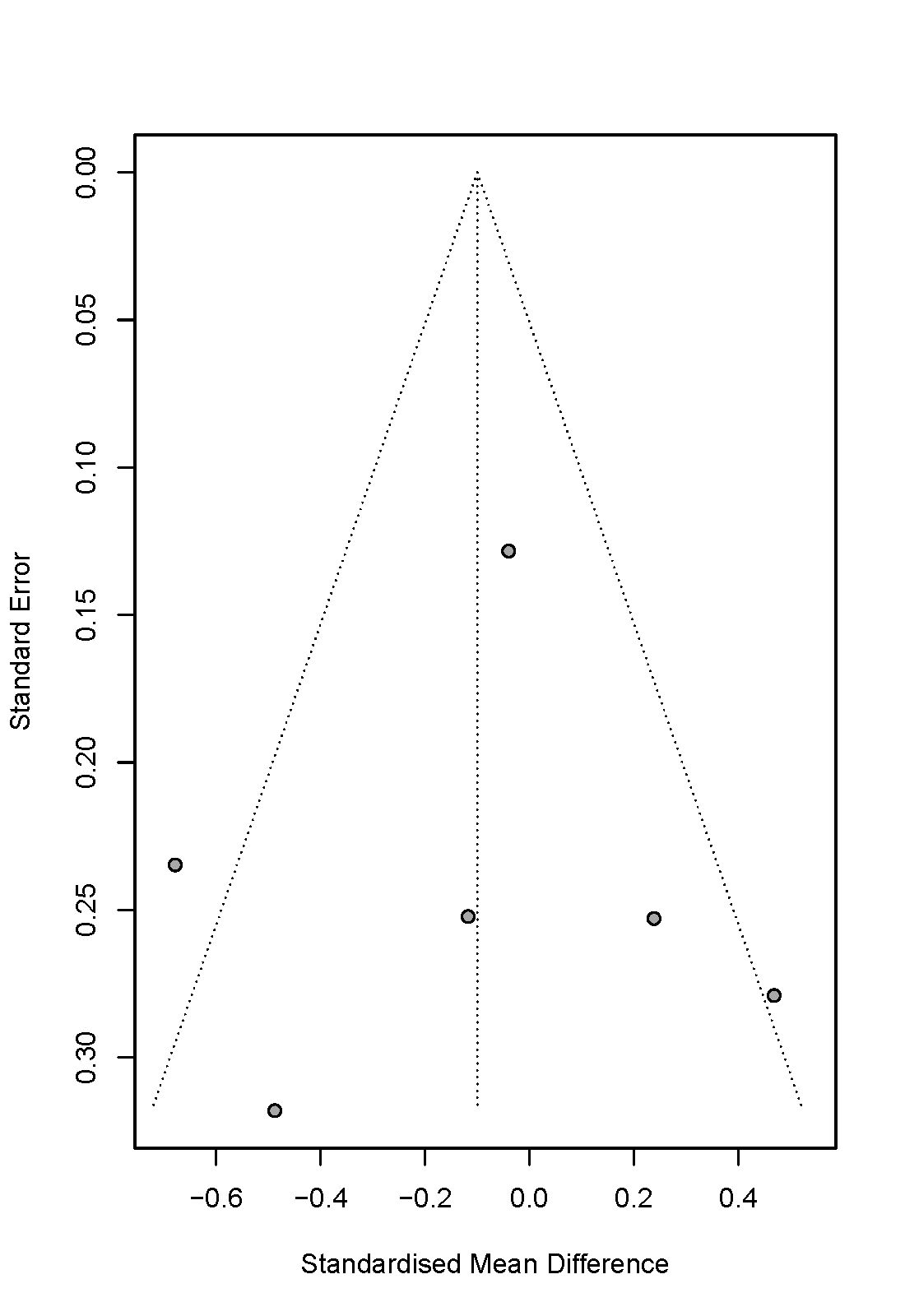
**

**S Fig 9.6 Funnel plot of ω-3 PUFAs and VWF%**

**
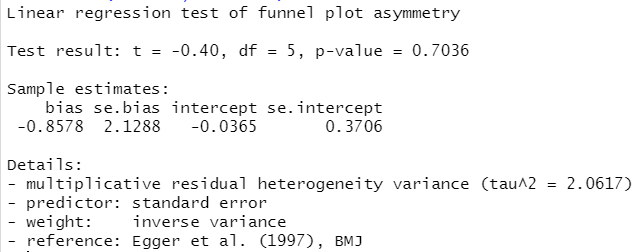
**

**S Fig 10.1 Peter’s bias of ω-3 PUFAs supplementation and coronary atherosclerotic plaques.**

**
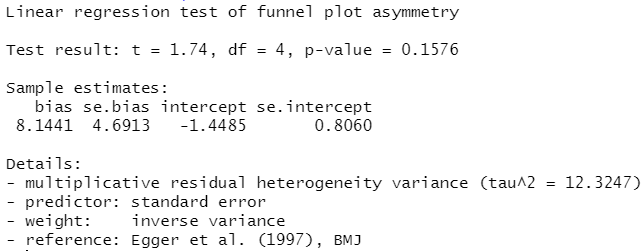
**

**S Fig 10.2 Peter’s bias of ω-3 PUFAs and most stenotic segment of the coronary arteries.**

**
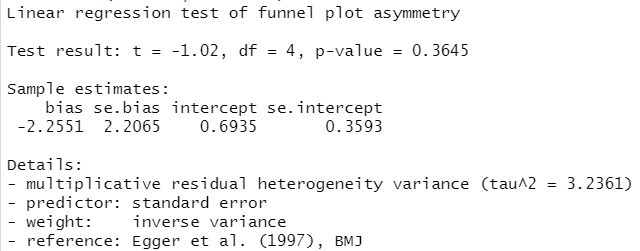
**

**S Fig 10.3 Peter’s bias of ω-3 PUFAs and fiber plaques volume**

**
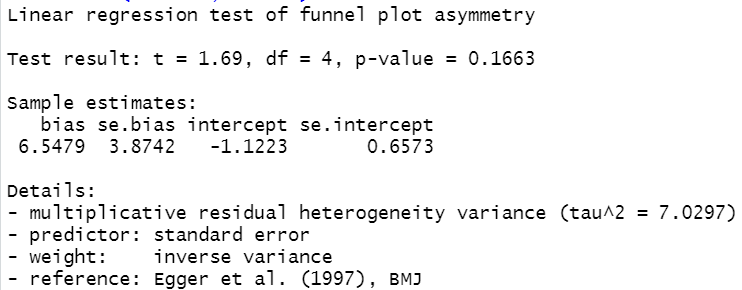
S Fig 10.4 Peter’s bias of ω-3 PUFAs and calcified plaque volume**

**
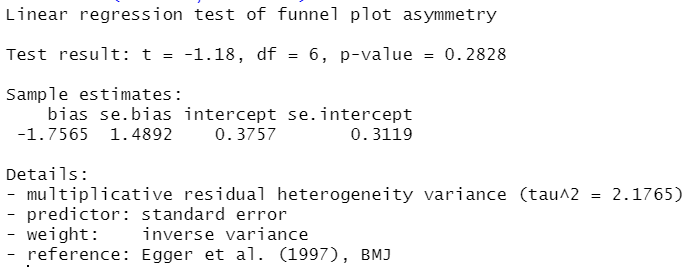
S Fig 10.5 Peter’s bias of ω-3 PUFAs and sVCAM-1.**

**
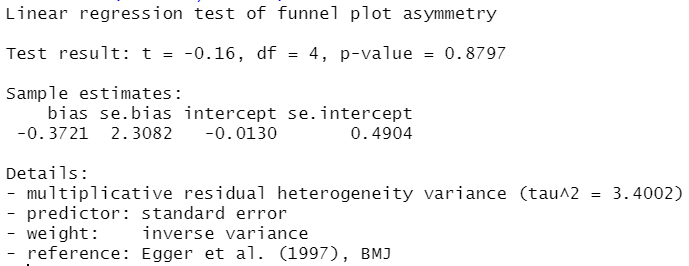
S Fig 10.6 Peter’s bias of ω-3 PUFAs and VWF%.**


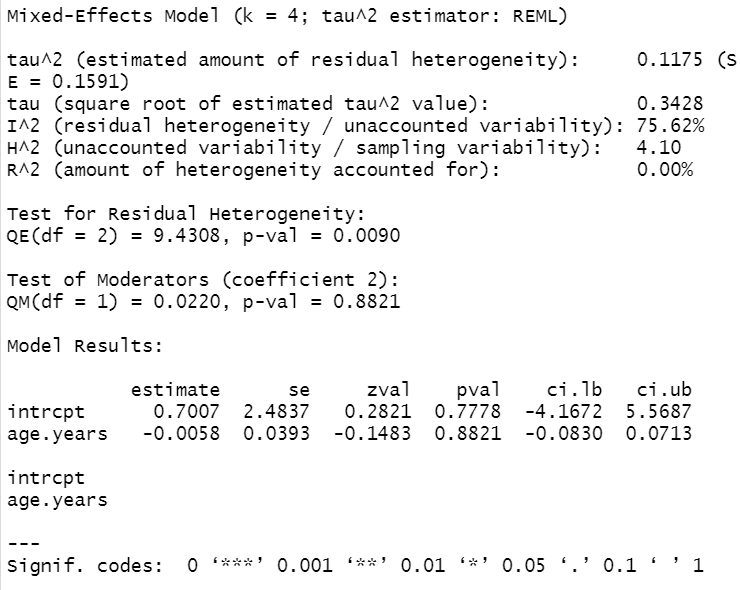
 **S Fig 11.1 R work station of meta-regression of individuals’ main age and the diameter of the narrowest segments of coronary arteries**


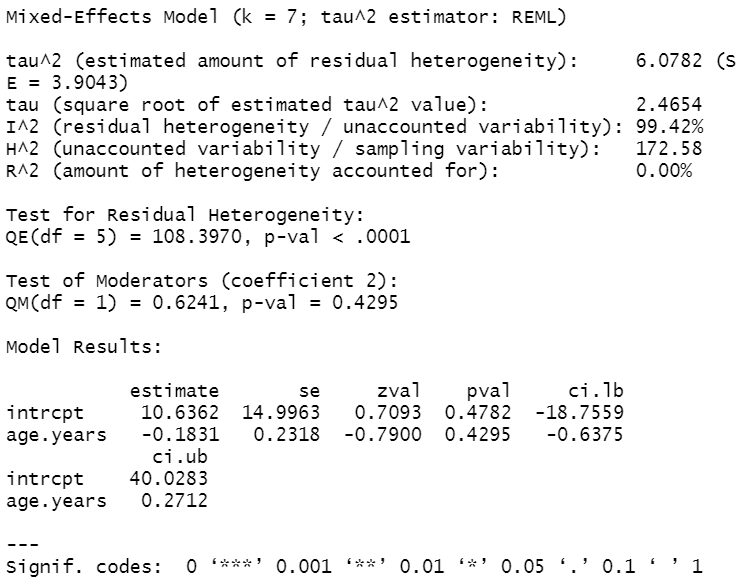
 **S Fig 11.2 R work station of meta-regression of individuals’ main age and volume of lipid plaques**


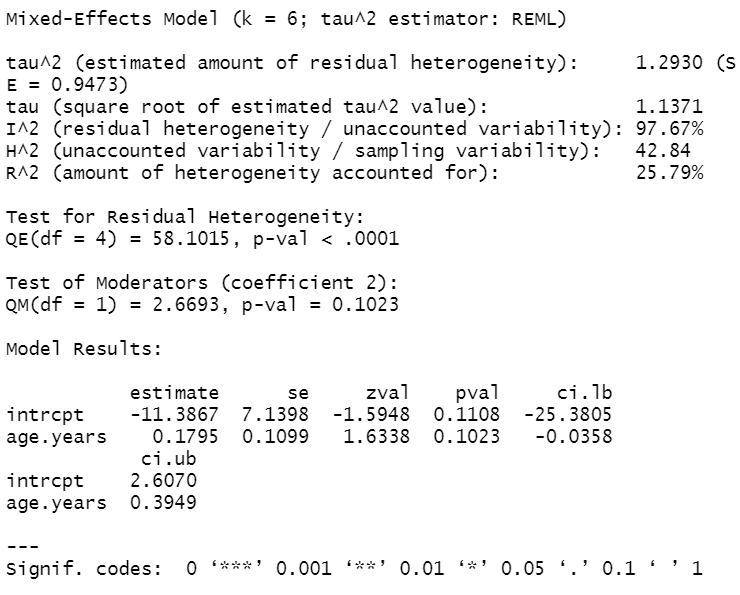
 **S Fig 11.3 R work station of meta-regression of individuals’ main age and volume of fiber plaques**


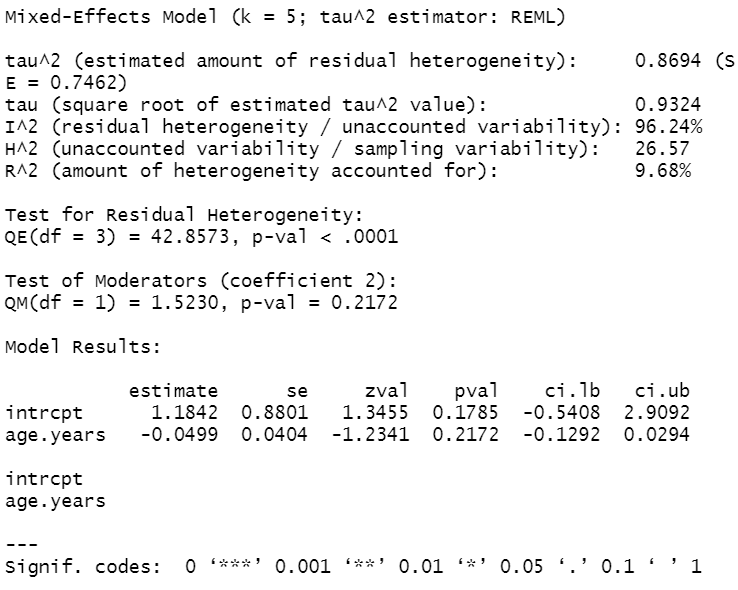
 **S Fig 11.4 R work station of meta-regression of individuals’ main age and volume of calcified plaques**


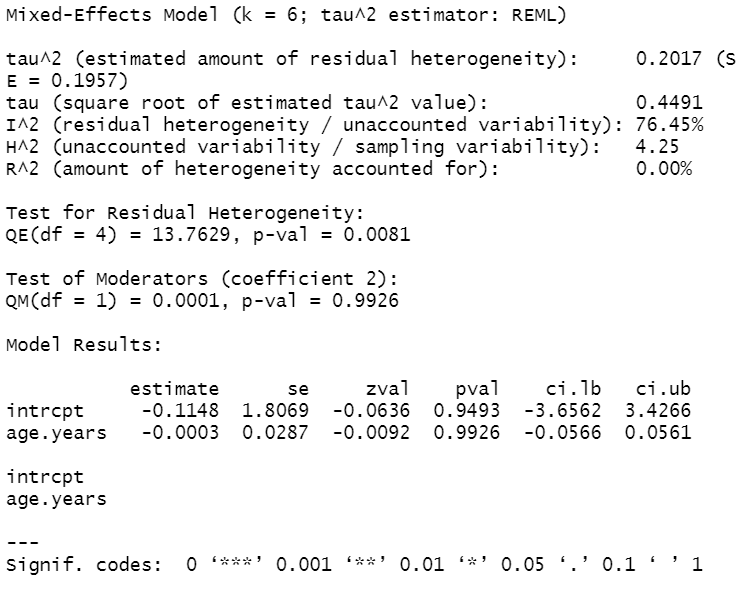
 **S Fig 11.5 R work station of meta-regression of individuals’ main age and volume of sVCAM-1 plaques**


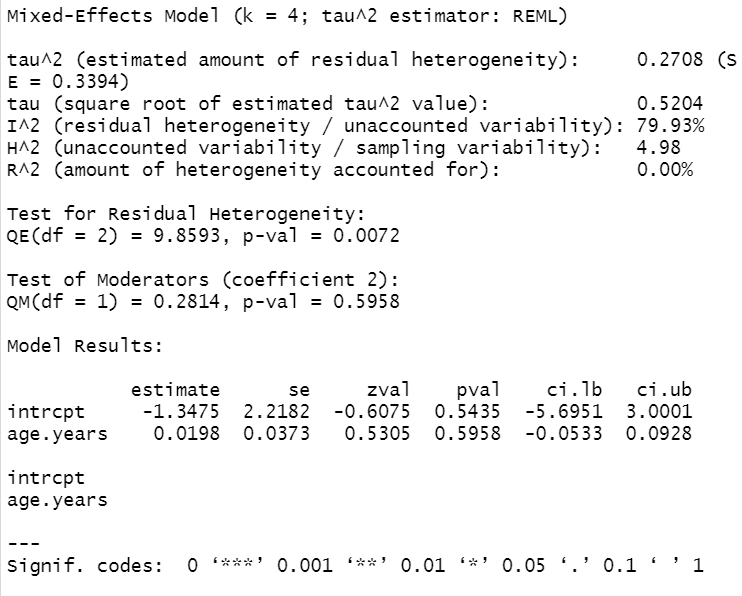
 **S Fig 11.6 R work station of meta-regression of individuals’ main age and volume of VWF% plaques**


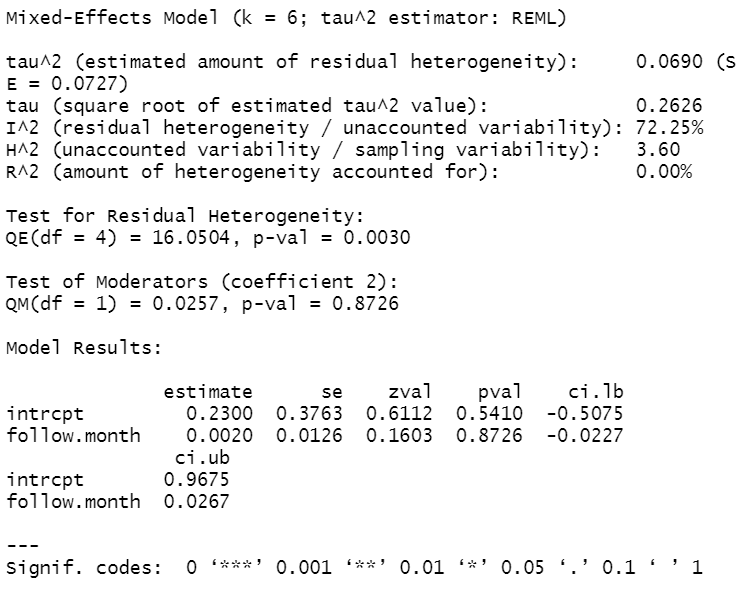
 **S Fig 11.7 R work station of meta-regression of follow-up duration and the diameter of the narrowest segments of coronary arteries**


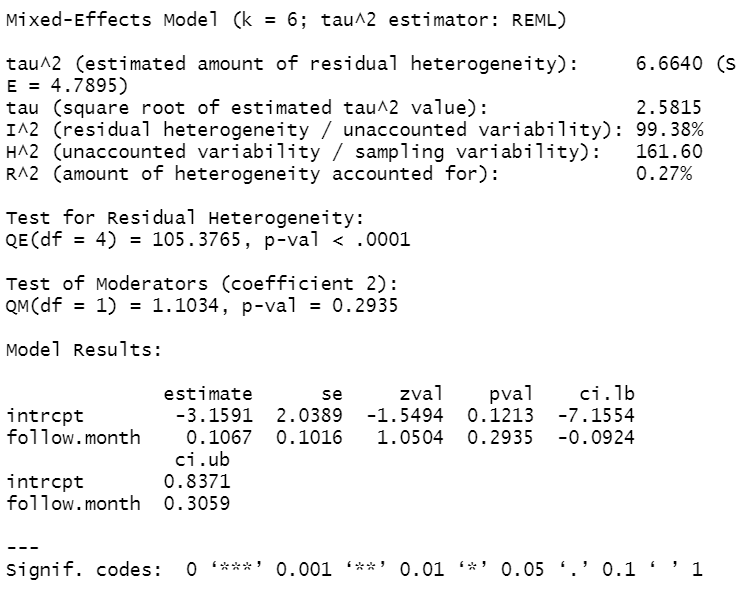
 **S Fig 11.8 R work station of meta-regression of follow-up duration and volume of lipid plaques**


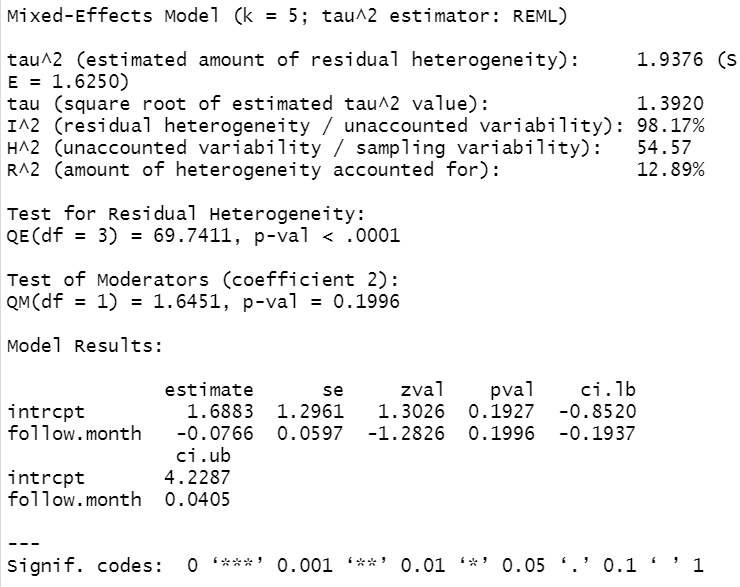
 **S Fig 11.9 R work station of meta-regression of follow-up duration and volume of fiber plaques**


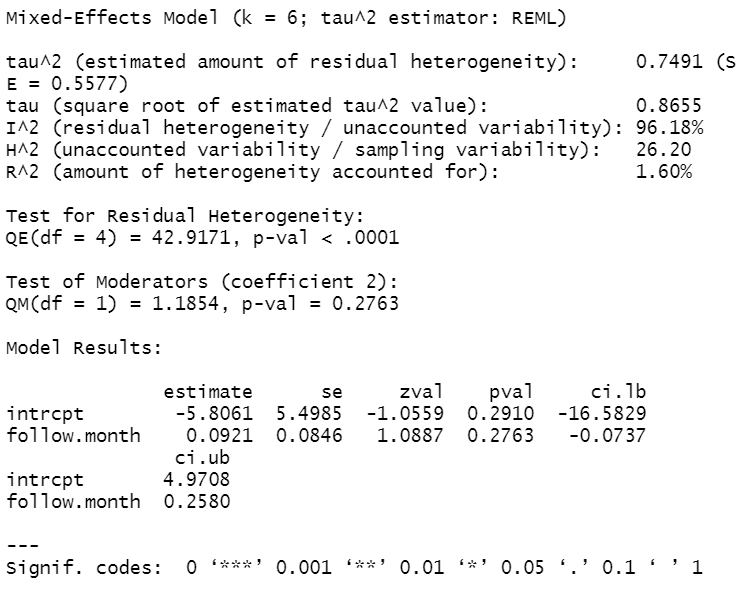
 **S Fig 11.10 R work station of meta-regression of follow-up duration and volume of calcified plaques**


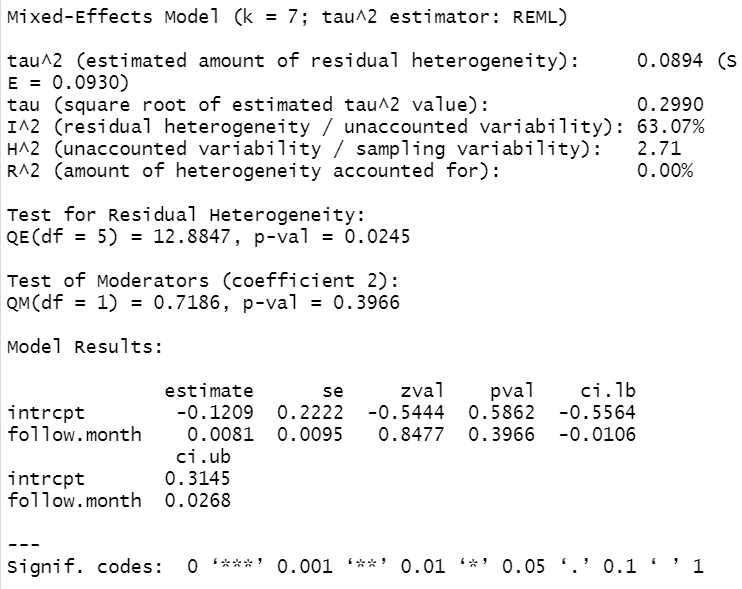
 **S Fig 11.11 R work station of meta-regression of follow-up duration and volume of sVCAM-1**


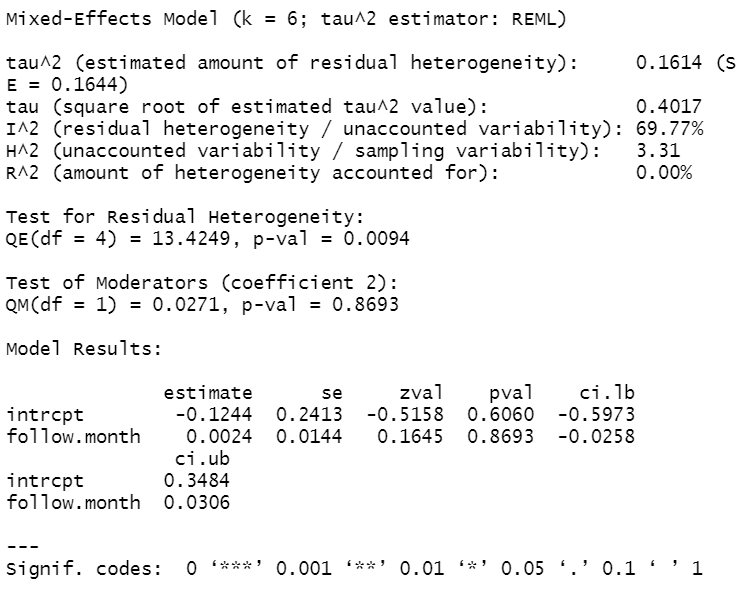
 **S Fig 11.12 R work station of meta-regression of follow-up duration and volume of lipid VWF%**


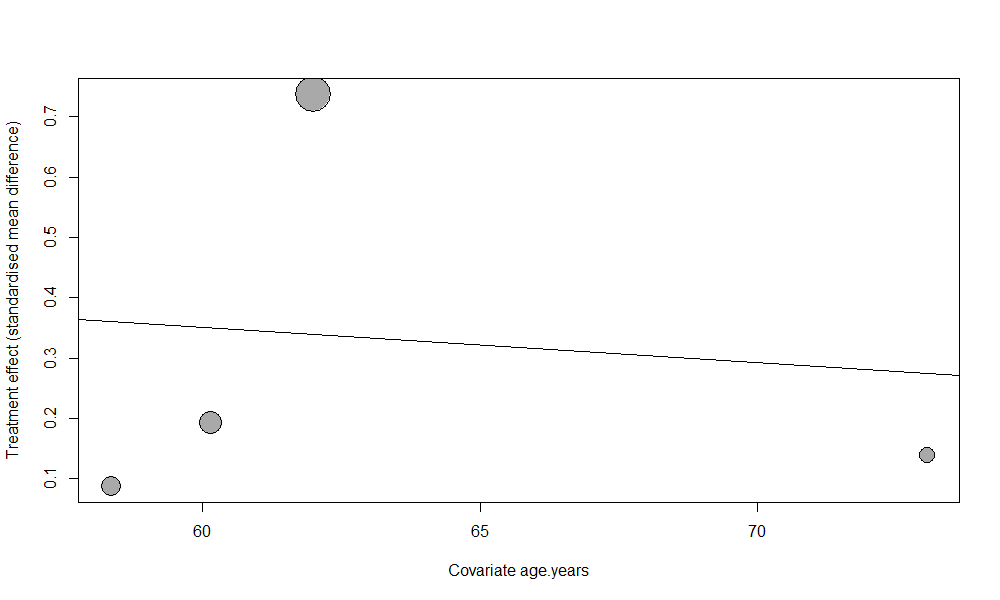
 **S Fig 12.1 Meta-regression curve of individuals’ main age and the diameter of the narrowest segments of coronary arteries**


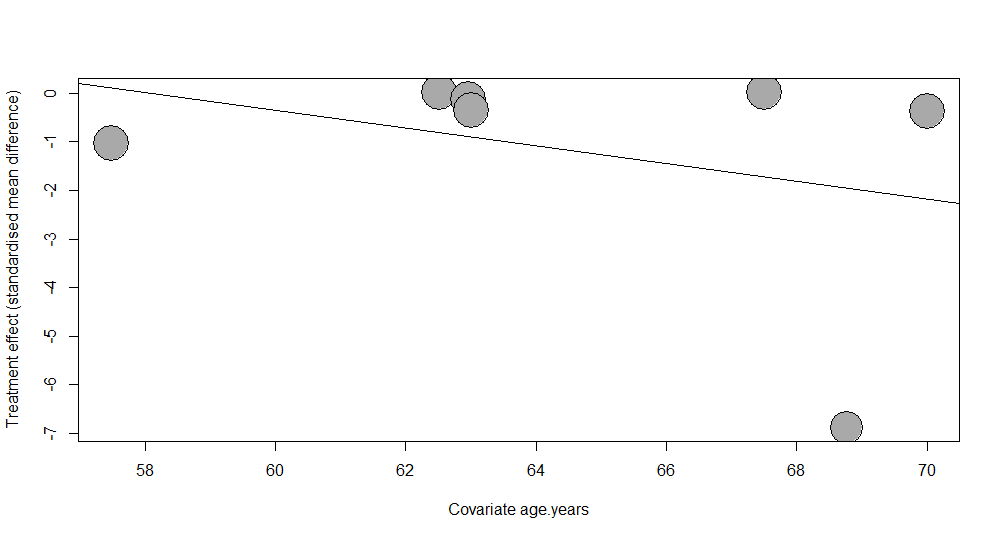
 **S Fig 12.2 Meta-regression curve of individuals’ main age and the volume of the lipid plaques**


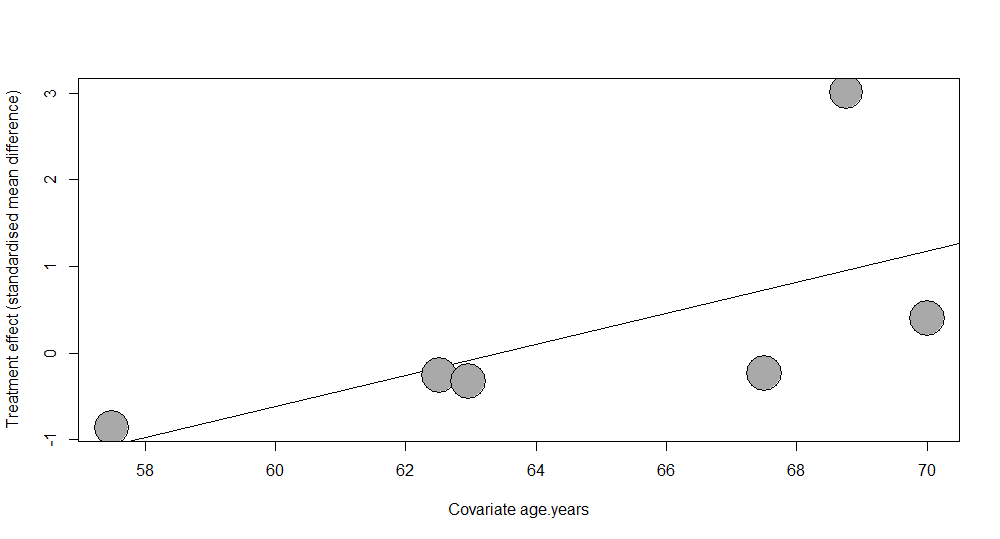
 **S Fig 12.3 Meta-regression curve of individuals’ main age and the volume of the fiber plaques**


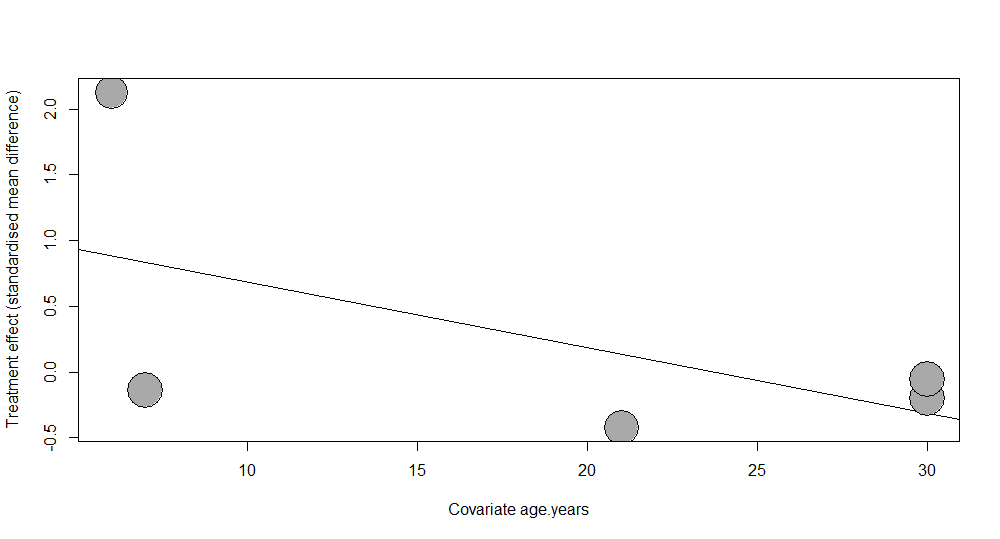
 **S Fig 12.4 Meta-regression curve of individuals’ main age and the volume of the calcified plaques**


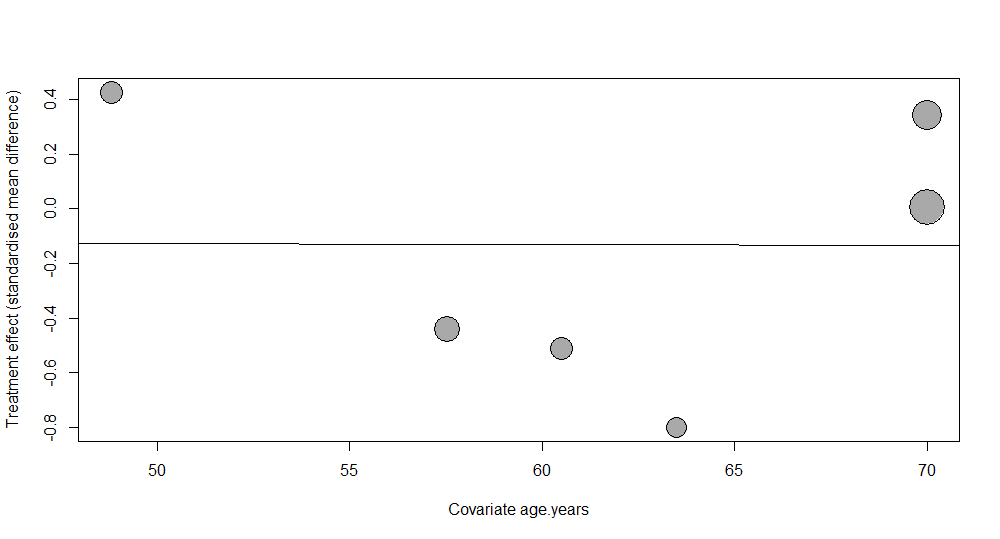
 **S Fig 12.5 Meta-regression curve of individuals’ main age and sVCAM-1**


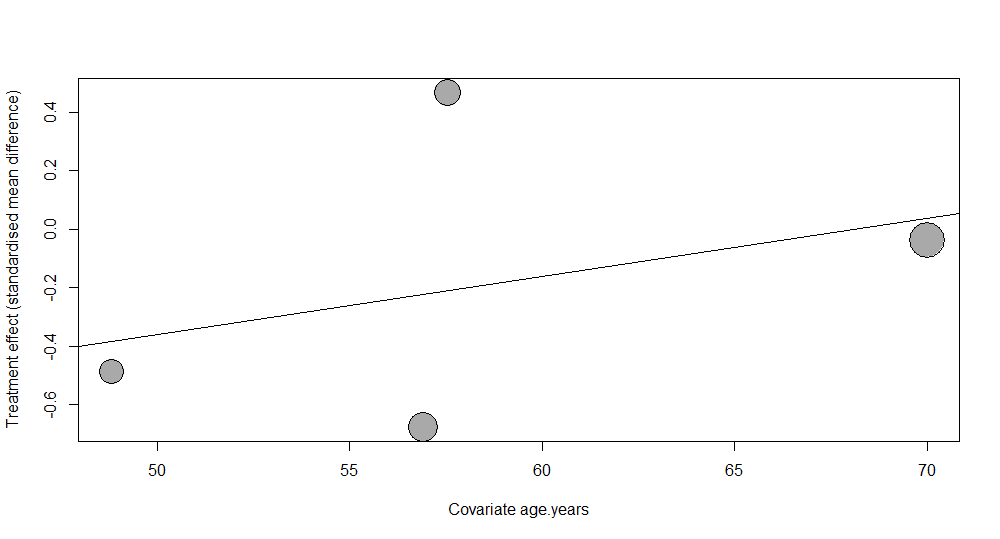
 **S Fig 12.6 Meta-regression curve of individuals’ main age and VWF%**


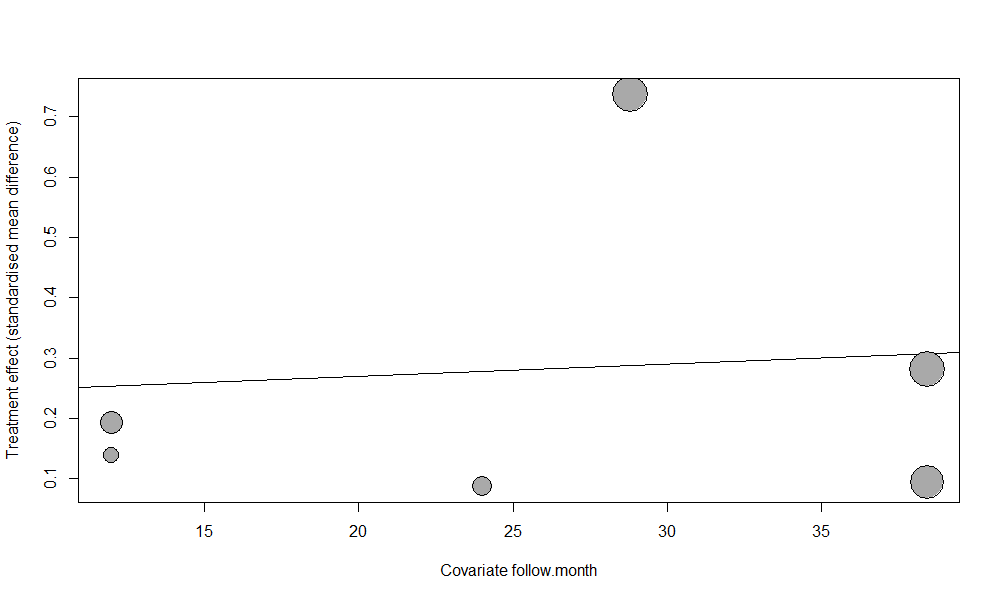
 **S Fig 12.7 Meta-regression curve of duration of follow-up and the diameter of the narrowest segments of coronary arteries**


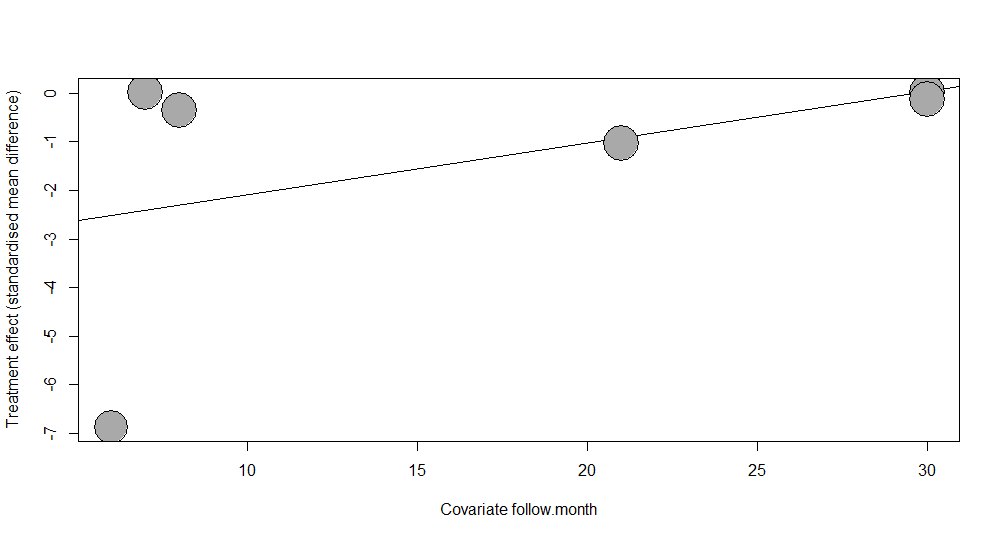
 **S Fig 12.8 Meta-regression curve of duration of follow-up and the volume of lipid plaques**


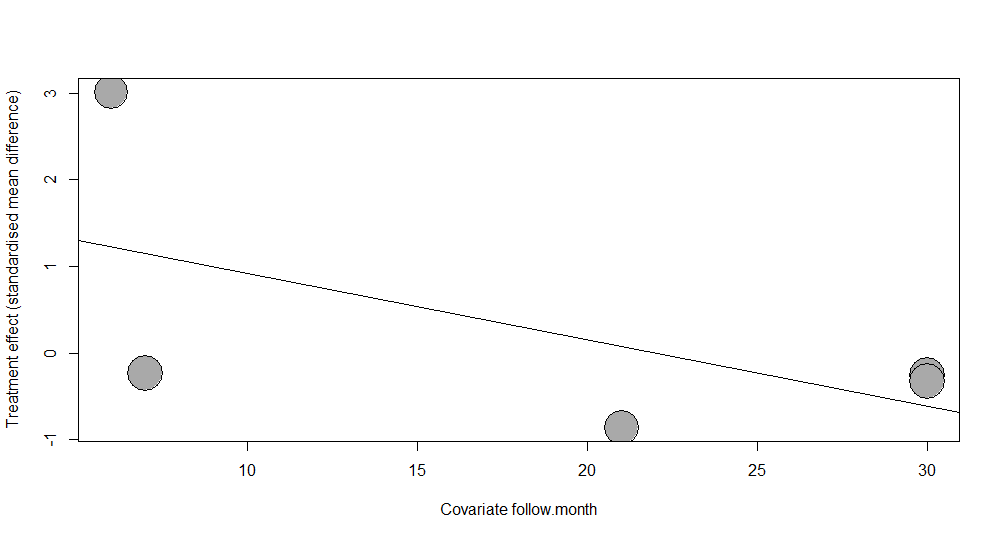
 **S Fig 12.9 Meta-regression curve of duration of follow-up and the volume of fiber plaques**


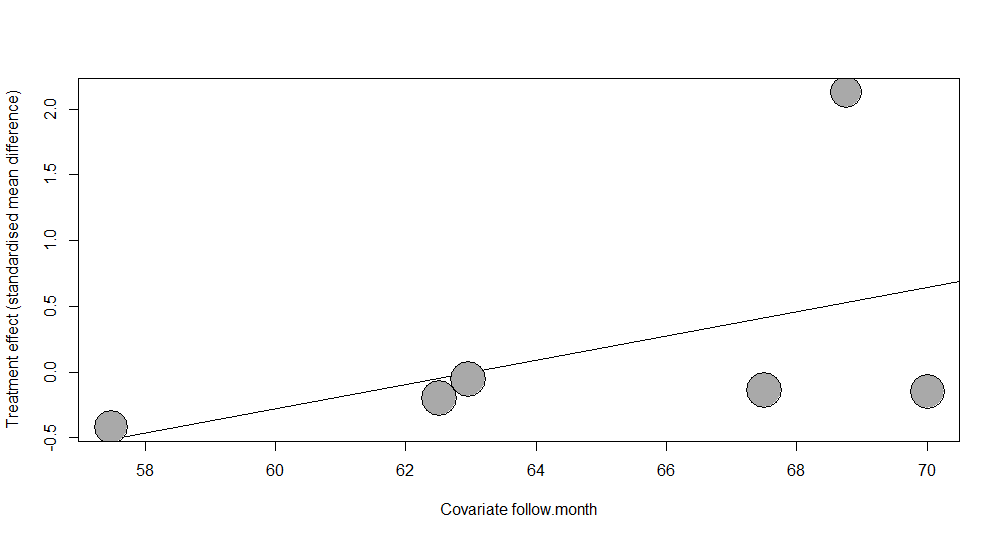
 **S Fig 12.10 Meta-regression curve of duration of follow-up and the volume of calcified plaques**


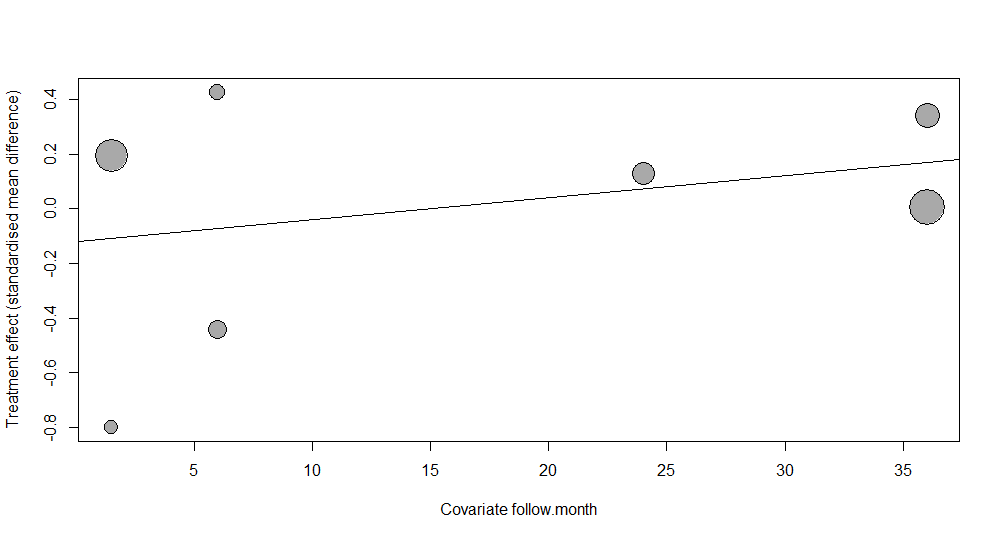
 **S Fig 12.11 Meta-regression curve of duration of follow-up and sVCAM-1**


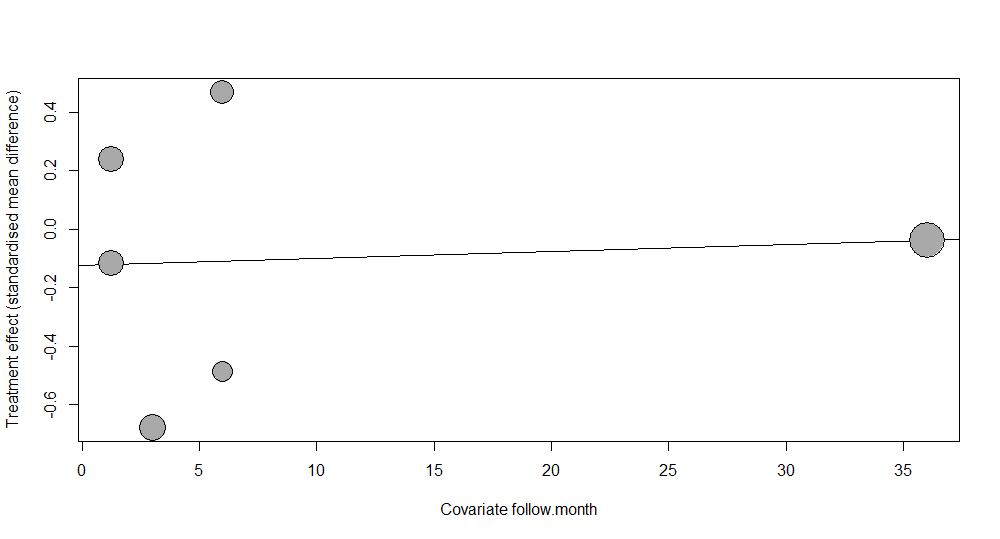
 **S Fig 12.12 Meta-regression curve of duration of follow-up and VWF%**

**S Appendix 1 Search strategy.**

| Search strategy | |
| --- | --- |
| No. | **Items** |
| #1 | Atheromatous Plaques【MeSh】 |
| #2 | Fatty Streak, Arterial【Title/Abstruct】 |
| #3 | Arterial Fatty Streak【Title/Abstruct】 |
| #4 | Arterial Fatty Streaks【Title/Abstruct】 |
| #5 | Fatty Streaks, Arterial【Title/Abstruct】 |
| #6 | Streak, Arterial Fatty【Title/Abstruct】 |
| #7 | Streaks, Arterial Fatty【Title/Abstruct】 |
| #8 | Fibroatheroma【Title/Abstruct】 |
| #9 | Fibroatheromas【Title/Abstruct】 |
| #10 | Fibroatheromatous Plaques【Title/Abstruct】 |
| #11 | Fibroatheromatous Plaque【Title/Abstruct】 |
| #12 | Plaque, Fibroatheromatous【Title/Abstruct】 |
| #13 | Plaques, Fibroatheromatous【Title/Abstruct】 |
| #14 | Atheroma【Title/Abstruct】 |
| #15 | Atheromas【Title/Abstruct】 |
| #16 | Atheromatous Plaque【Title/Abstruct】 |
| #17 | Plaque, Atheromatous【Title/Abstruct】 |
| #18 | Plaques, Atheromatous【Title/Abstruct】 |
| #19 | Atheroscleroses【Title/Abstruct】 |
| #20 | Atherogenesis【Title/Abstruct】 |
| #21 | #1 or #2 or #3 or #4 or #5 or #6 or #7 or #8 or #9 or #10 or #11 or #12 or #13 or #14 or #15 or #16 or #17 or #18 or #19 or #20 |
| #22 | Blood Vessel【MeSh】 |
| #23 | Vessel, Blood【Title/Abstruct】 |
| #24 | vascular【Title/Abstruct】 |
| #25 | Vessels, Blood【Title/Abstruct】 |
| #26 | Vessels【Title/Abstruct】 |
| #27 | #24 or #25 or #26 |
| #28 | Coronary Heart Disease【MeSh】 |
| #29 | Coronary Diseases【Title/Abstruct】 |
| #30 | Disease, Coronary【Title/Abstruct】 |
| #31 | Diseases, Coronary【Title/Abstruct】 |
| #32 | Coronary Heart Diseases【Title/Abstruct】 |
| #33 | Disease, Coronary Heart【Title/Abstruct】 |
| #34 | Diseases, Coronary Heart【Title/Abstruct】 |
| #35 | Heart Disease, Coronary【Title/Abstruct】 |
| #36 | Heart Diseases, Coronary【Title/Abstruct】 |
| #37 | Acids, Unsaturated Fatty【Title/Abstruct】 |
| #38 | #28 or #29 or #30 or # 31 or #32 r #33 or #34 or #35 or #36 or #37 |
| #39 | Unsaturated Fatty Acids【MeSh】 |
| #40 | Acids, Unsaturated Fatty【Title/Abstruct】 |
| #41 | Unsaturated Fatty Acid【Title/Abstruct】 |
| #42 | Acid, Unsaturated Fatty【Title/Abstruct】 |
| #43 | Fatty Acid, Unsaturated【Title/Abstruct】 |
| #44 | Polyunsaturated Fatty Acids【Title/Abstruct】 |
| #45 | Acids, Polyunsaturated Fatty【Title/Abstruct】 |
| #46 | Fatty Acids, Polyunsaturated【Title/Abstruct】 |
| #47 | Polyunsaturated Fatty Acid【Title/Abstruct】 |
| #48 | Acid, Polyunsaturated Fatty【Title/Abstruct】 |
| #49 | Fatty Acid, Polyunsaturated【Title/Abstruct】 |
| #50 | #39 or #40 or #41 or #42 or #43 or #44 or #45 or #46 or #47 or #48 or #49 |
| #51 | ω-3【MeSh】 |
| #52 | n-3【Title/Abstruct】 |
| #53 | Omega-3【Title/Abstruct】 |
| #54 | #51 or #52 or #53 |
| #55 | # 54 and #50 |
| #56 | #21 or #27 |
| #57 | #55 and #56 |
